# Targeting of PTP4A3 overexpression sensitises HGSOC cells towards chemotherapeutic drugs

**DOI:** 10.1002/1878-0261.70092

**Published:** 2025-07-14

**Authors:** Ana López‐Garza, David James, Emma Creagh, James T. Murray

**Affiliations:** ^1^ School of Biochemistry and Immunology Trinity Biomedical Sciences Institute, Trinity College Dublin Dublin 2 Ireland; ^2^ Swansea University Medical School Swansea University Swansea UK; ^3^ Present address: Vall d'Hebron Institute of Oncology Barcelona Spain

**Keywords:** autophagy, chemoresistance, ovarian cancer, PTP4A3

## Abstract

Ovarian cancer (OC) has the highest mortality rate of all gynaecological malignancies, partly attributable to its propensity for chemotherapy resistance. The most common subtype of OC is serous, of which high‐grade serous ovarian cancer (HGSOC) is the most lethal subtype. Protein tyrosine phosphatase 4A3 (PTP4A3) overexpression is implicated in tumour cell invasion and metastasis by upregulating the PI3K/Akt/mTORC1 axis. Previously, we reported that PTP4A3 increased the survival of non‐serous OC cells by activating the autophagy pathway. Here, we investigated the impact of PTP4A3 on cell proliferation, autophagy and chemoresistance in HGSOC cells and whether targeting PTP4A3 in HGSOC cells that overexpress this phosphatase would sensitise them to existing chemotherapeutic drugs. Gene silencing of PTP4A3 resulted in the upregulation of compensatory mechanisms that overcame the loss of PTP4A3 expression, but this was mitigated by pan‐PTP4A inhibition with JMS‐053 in HGSOC cells. Moreover, shRNA‐mediated silencing of PTP4A3 sensitised HGSOC cells to clinically relevant chemotherapeutic drugs. Overall, we show that compensatory mechanisms from PTP4A1 and PTP4A2 can arise when specifically targeting PTP4A3 in HGSOC and that pan‐PTP4A inhibition can overcome those effects.

## Graphical abstract

In HGSOC with normal KRAS expression, high PTP4A3 expression regulates autophagy activation. Conversely, in HGSOC with high KRAS expression, KRAS dictates autophagy control, and PTP4A3 is not required. When high PTP4A3 expression is inhibited, HGSOC cells are preferentially sensitised towards DNA‐damaging agents. Thus, targeting PTP4A3 when highly expressed in OC may increase sensitivity to existing chemotherapy drugs.

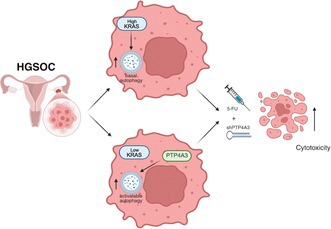

**Abbreviations**
HGSOC: high‐grade serous ovarian cancer
PTP4A: protein tyrosine phosphatase 4A
shRNA: short hairpin RNA

## 1 Introduction

Ovarian cancer (OC) is the most lethal gynaecologic malignancy and the fifth leading cause of cancer‐related deaths among women. Although it is less common than breast cancer, the fatality rate of OC is three times higher, with a 5‐year survival rate of 46.5% [1, 2, 3]. This high mortality rate is attributed to poor early detection diagnostic methods, inadequate therapeutic options, high recurrence rates and its propensity for drug resistance [1, 2]. Due to its limited detection at early stages, four in every five women are diagnosed when the cancer has already metastasised to other tissues [1, 2, 3]. Serous is the most common subtype of OC and is subdivided into high‐ and low‐grade serous ovarian cancer (HGSOC and LGSOC). Two‐thirds of ovarian cancer mortality is specifically due to the HGSOC subtype [2].

The prenylated protein tyrosine phosphatase (PTP), PTP4A3, is one of three PTPs (PTP4A1–3, also known as phosphatase of regenerating liver 1–3) that share >75% sequence identity [4, 5]. Ordinarily, PTP4A3 is involved in the early development of the circulatory system, with expression detected in the foetal heart, developing blood vessels and pre‐erythrocytes, but not in adult tissues [6]. During cancer, PTP4A3 transcriptional upregulation of mRNA correlates with advanced stage and poor prognosis [7]. Transcriptional PTP4A3 upregulation and protein expression, which can occur via p53, has been implicated in tumour cell invasion and metastasis, mediated through its ability to disrupt cell adhesion and enhance migratory signalling pathways [6, 8]. PTP4A3 protein is also involved in the regulation of the epithelial‐mesenchymal transition (EMT), through its inhibition of PTEN and activation of PI3K/AKT, MAPK/ERK, and SRC signalling [6, 9]. The phosphatase of PTP4A3 remains poorly understood; however, it is crucial for its function since the inactivating mutant C104S is unable to activate these pathways, inferring that PTP4A3 is at least basally active [10]. Recent studies report a physiological role for PTP4A3 and 1 protein in regulating micropinocytosis via its lipid phosphatase activity, suggesting that tumour cells may exploit this mechanism to survive in nutrient‐deprived microenvironments [11]. Moreover, research from the Zeng group has shown that PTP4A3 antigens are externalised by tumour cells, making them accessible for the first‐in‐class humanised antibody against PTP4A3, PRL3‐zumab, which is in Phase 2 trials (NCT04452955) [12, 13]. Studies have demonstrated that the PTP4A3 antibody therapy *in vivo* specifically inhibits PRL3‐expressing, but not PRL3 null, tumours [12].

Although the involvement of PTP4A3 in diseases other than cancer remains largely unexplored, it has also been identified as a potential protein biomarker and therapeutic target in Alzheimer's disease, implicated in a contributory role in the disruption of the blood–brain barrier [14].

Autophagy is a cellular catabolic process by which cytoplasmic material is ultimately degraded by the lysosome. It functions as a homeostatic cellular recycling mechanism to ensure cellular survival by minimising the accumulation of cellular damage. Autophagy is initiated when cells are confronted with potentially dangerous environmental, physical, chemical or metabolic signals, such as thermal stress, irradiation, changes in pH/osmolality or a shortage in nutrients and/or oxygen [15, 16, 17]. Dysfunctional autophagy is associated with numerous human diseases, including diabetes, response to infection, neurodegeneration and cancer [18, 19].

Autophagy plays a complex role in cancer development and progression that depends on the phase of carcinogenesis and the tumour context. Drugs that induce or inhibit autophagy have both been described to have anticancer effects [15, 20]. Autophagy can limit genomic instability and tumour development by protecting cells from ROS‐induced DNA and proteotoxicity [21] and chronic upregulation of autophagy is associated with the induction of cell death [15, 20]. In contrast, upregulation of autophagy in growing tumours can compensate for limited nutrient supply and mitigate genotoxic and metabolic stresses. This is underscored in some cancers, where the KRAS oncogene can drive autophagy addiction [22, 23, 24, 25]. The pro‐tumourigenic activity of autophagy is also associated with drug resistance and represents a target for combinatorial therapeutic approaches [18, 26]. Anticancer therapies that target autophagy are dependent on actual levels of ongoing autophagy in tumour cells [27, 28].

PTP4A3 protein expression is upregulated in multiple human cancers, including OC [15, 29]. Overexpression of PTP4A3 mRNA in OC has been detected in advanced stage‐III when compared to early stage‐I tumours, suggesting a correlation between PTP4A3 mRNA and protein expression and the invasiveness of OC [30, 31]. Our previous study demonstrated that PTP4A3 protein expression increases the survival of ovarian cancer cells *in vitro* by activating the autophagy pathway. High PTP4A3 mRNA expression was shown to positively correlate with the expression of two critical autophagy genes, PIK3C3 and BCLN1, which significantly predict poorer OC prognosis in patients, suggesting a critical role of autophagy in PTP4A3‐driven OC progression [15, 20].

The present study focused on understanding the impact of PTP4A3 protein on cell growth, proliferation and autophagy mechanisms in OC cells. In particular, we sought to understand how PTP4A3 regulates autophagy in HGSOC cells and whether targeting PTP4A3 in cancer cells that overexpress this protein sensitises them to chemotherapeutic drugs.

## 2 Materials and methods

### 2.1 Cell culture

Three HGSOC cell lines used in this study, OVCAR 3 (RRID: CVCL_0465), OVCAR 4 (RRID: CVCL_1627) and Kuramochi (RRID: CVCL_1345), authenticated by genotyping, were kindly provided by Dr. Nuala McCabe (The Queen's University, Belfast). The OVCAR 3 cell line was established from the malignant ascites of a 60‐year‐old Caucasian patient with progressive adenocarcinoma of the ovary, after combination chemotherapy with cyclophosphamide, adriamycin and cisplatin [32, 33]. The OVCAR 4 cell line was established from the malignant ascites of a 42‐year‐old Caucasian patient with high‐grade ovarian cancer [34, 35, 36]. The Kuramochi cell line was established from the malignant ovarian cancer of an unspecified‐age Japanese woman [35, 36]. All three cell lines originate from the metastatic site and possess TP53 homozygous sequence variations: p.Arg248Gln, p.Leu130Val and p.Asp281Tyr, respectively. Lines were maintained in RPMI‐1640 (Sigma‐Aldrich, UK R8758) supplemented with 10% (v/v) Foetal Bovine Serum (Sigma‐Aldrich F7524), 1% (w/v) L‐glutamine (Sigma‐Aldrich G7513) and 1% (w/v) Penicillin/Streptomycin (Sigma‐Aldrich P4333) at 37 °C, 95% humidity and 5% CO_2_. Cell lines were used at passages from 15 to 40, for up to 10–15 passages each of them before thawing a new vial. They were routinely confirmed as mycoplasma‐free by PCR screening.

### 2.2 Stable knockdown cell line generation

Replication‐defective lentiviral particles were generated using HEK293T cells PEI transfected with 4.5 μg pCMV‐DVPR (packaging plasmid), 1.5 μg pCMV‐VSV‐G (envelope plasmid) and 6 μg pLKO.1 (TRCN000001883 PTP4A3 MISSION shRNA, or scramble control plasmid, Sigma‐Aldrich). Media containing lentiviral particles was harvested after 48 h, filtered through a 0.45 μm sterile filter and then added to target cells. Selection of PTP4A3 silenced Kuramochi and OVCAR 4 cells was performed with 1.6 and 0.8 μg·mL^−1^ puromycin, respectively. Knockdown efficiency was validated by western immunoblot.

### 2.3 Transient protein overexpression

pEGFPC1 parent plasmids (Clontech, Oxford, UK) were further cloned in‐house to generate PTP4A3 expressing (9). Plasmids were amplified using chemically competent NEB5α *E. coli* cells (New England Biolabs, Hitchin, UK). The cDNA plasmids were isolated using a HiSpeed Plasmid Maxi Kit (QIAGEN, Manchester, UK). Once eluted, the concentration (A280 nm) and purity (A260/A280 nm) of plasmid DNA were assessed using a NanoDrop spectrophotometer ND‐1000. To overexpress a protein of interest, target cells were PEI transfected with 4.6 μg cDNA. Transfection efficiency was assessed by fluorescence microscopy and western immunoblot.

### 2.4 Cell growth and death monitoring

Cells were plated in 96‐well plates before adding increasing doses of 5‐fluorouracil (Sigma‐Aldrich, 0–1 mm in DMSO), cisplatin (Sigma‐Aldrich, 0–40 μm in 0.9% NaCl), paclitaxel (Sigma‐Aldrich, 0–100 nm in DMSO) or JMS‐053 (Clinisciences, Nanterre, France (formerly Generon), cat# AOB31947, 0–25 μm in DMSO) together with 2.5 μg·mL^−1^ propidium iodide (PI) to stain dead cells. Plates were transferred to the Incucyte S3 and the system was set to take 4 images per well every 3 h for 3 d. Confluency and the number of PI‐positive cells were quantified using the Incucyte software and presented using GraphPad Prism 9 software.

### 2.5 Western blot analysis

Whole‐cell protein lysates were sonicated using a MicroTip sonicator (20% output, for 10 s), and protein concentration was quantified using a Bradford protein quantification assay (Thermo‐Pierce). Lysates were separated by SDS/PAGE, transferred onto PVDF membranes and incubated in blocking buffer of 5% (w/v) non‐fat dried milk (NFDM) in tris‐buffered saline, 0.1% (v/v) Tween 20 (TBS‐T) for 30 min at room temperature. Membranes were incubated overnight at 4 °C on a rocker with the following antibodies in blocking buffer: β‐actin (A5441; Sigma‐Aldrich, Gillingham, UK), LC3B (2775; Cell Signaling Technology, Leiden, Netherlands), S6K1 (9202), p‐S6K1 (9205), ERK1/2 (9102), p‐ERK1/2 (9106), ULK1 (4773), p‐ULK1 (6888), p‐AKT (9271), PTP4A3 (sc‐130 355; Santa Cruz Biotechnology, Heidelberg, Germany), AKT [37], pan‐PTP4A3 (MAB32191; Bio‐Techn, Abingdon, UK) and GFP (A‐11122; Thermo Fisher Scientific, Horsham, UK), then washed thrice with TBS‐T and incubated with species‐appropriate HRP‐conjugated secondary antibodies (Thermo Fisher Scientific) diluted in blocking buffer. After three additional washes in TBS‐T, membranes were developed with ECL (Merck Millipore, Gillingham, UK 638173). The intensity of protein bands was quantified by densitometry using ImageLab software and presented using GraphPad Prism 10 software.

### 2.6 Statistical analysis

GraphPad Prism 10 software was employed for statistical analysis. All data are represented as mean ± SEM, with experiments done as *n* = 3, unless otherwise stated in the figure legend. Statistical significance was indicated when *P* < 0.05. A one‐way ANOVA test was used to compare two conditions. A two‐way ANOVA with a Tukey multiple comparisons test was used to compare more than two groups of individual treatments.

## 3 Results

### 3.1 PTP4A3 expression and PI3K and Ras activity in HGSOC cells

**Fig. 1:**
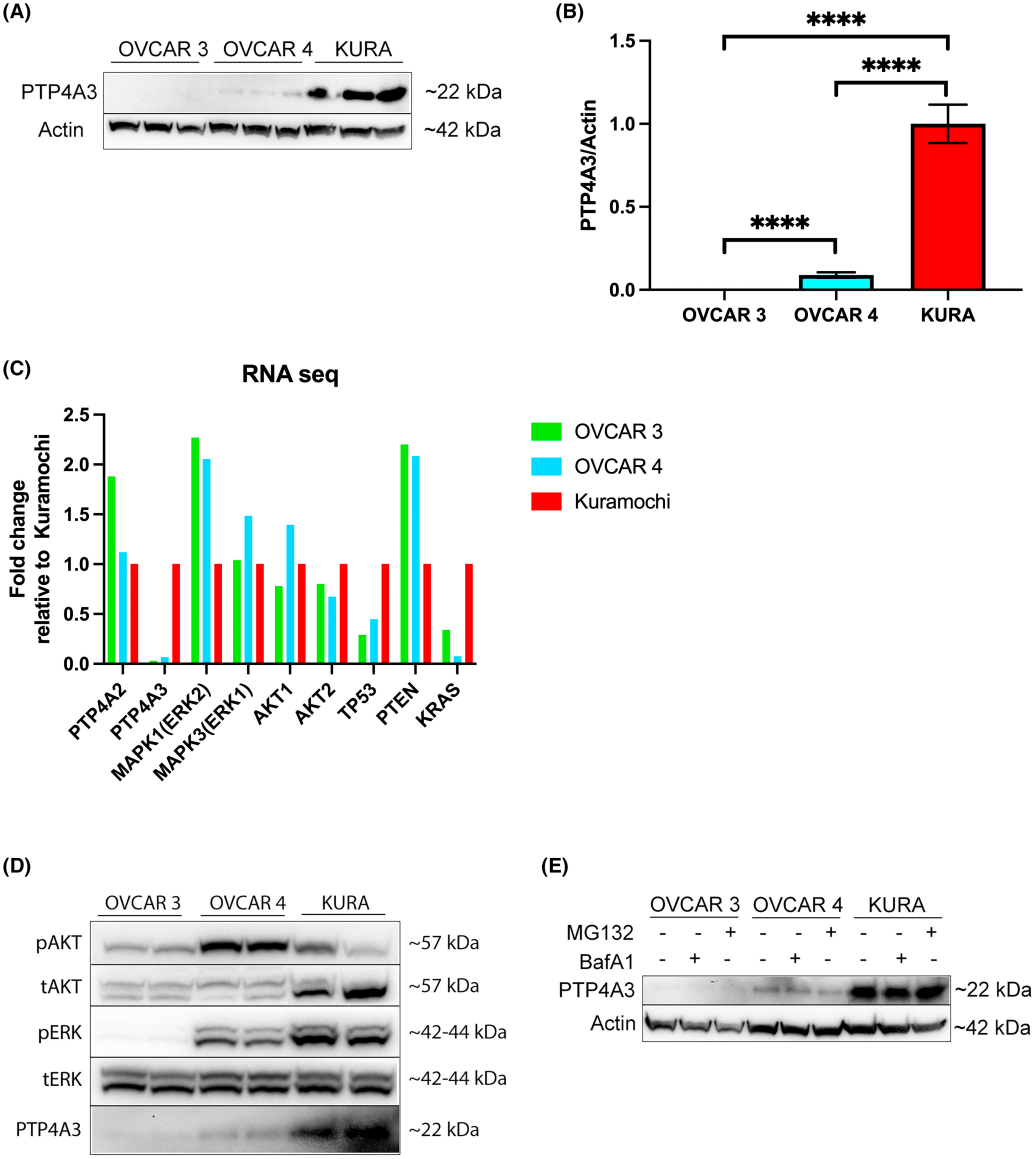
The expression of PTP4A3, PI3K and Ras signalling pathway proteins varies across HGSOC cell lines. (A) Expression of PTP4A3 was assessed in OVCAR 3, OVCAR 4 and Kuramochi cells by Western immunoblot, and (B) this was quantified by densitometry, with PTP4A3 normalised to β‐actin. (C) NCBI GEO repository RNA‐seq data was analysed for gene expression in the three HGSOC cell lines, normalised to Kuramochi levels. (D) Cell lysates were analysed for phosphorylation of AKT/PKB (pSer473) and ERK1‐2 (pThr202‐Tyr204). Antibodies that recognise total AKT/PKB and ERK1‐2 were used as controls. Expression of PTP4A3 is shown for comparison. (E) Turnover of PTP4A3 was analysed by incubating with or without either MG132 (2.5 μm, 2 h) or BafA1 (100 nm, 1 h) before lysates were collected, with β‐actin as loading control. Results are representative of at least three independent experiments. Error bars = ±SEM. ****P < 0.0001 by unpaired *t*‐test.

The expression of PTP4A3 protein was investigated in three HGSOC cell lines: Kuramochi, OVCAR 4 and OVCAR 3 [38]. There was negligible detection of PTP4A3 protein in OVCAR 3 cells, whereas Kuramochi cells expressed high levels, and OVCAR 4 cells expressed ~10‐fold less PTP4A3 than Kuramochi cells (Fig. 1A,B). We categorised these cell lines as having no (OVCAR 3), low (OVCAR 4) and high (Kuramochi) PTP4A3 expression for subsequent investigations.

PTP4A3 is implicated in both RAS and PI3K pathway signalling. Data collected from Cellosaurus and Harmonizome, combining CCLE, BioGPS and GDSC databases, revealed that OVCAR 3 cells express mutated PIK3R1 that affects the PI3K signalling pathway, while OVCAR 4 cells harbour mutations in MAP3K1 in the RAS pathway. Moreover, OVCAR 3 and Kuramochi also possess high KRAS mRNA expression, whereas OVCAR 4 cells have low mRNA expression of MEK2, suggesting dysregulation of RAS pathway signalling by opposing mechanisms. Finally, Kuramochi and OVCAR 3 cells have been shown to possess high and low PTP4A3 mRNA expression levels, respectively [39]. Analysis of RNA‐seq datasets, normalised to Kuramochi expression levels, was compared (Fig. 1C). PTP4A3 RNA expression in OVCAR 3 and OVCAR 4 was only 3% and 6.7%, respectively, of the amount expressed in Kuramochi, which correlated with PTP4A3 protein levels (Fig. 1A,B). Interestingly, PTP4A2 gene expression was almost 2‐fold higher in OVCAR 3 cells than in OVCAR 4 and Kuramochi cells (Fig. 1C). KRAS amplification in Kuramochi was also observed in the RNA‐seq datasets, with OVCAR 3 having 3‐fold less and OVCAR 4 having >12‐fold less KRAS expression than Kuramochi cells. In agreement with previous studies, the expression of TP53 correlated with PTP4A3 expression levels [40], whereas PTEN was inversely correlated with PTP4A3 expression [30] across the three cell lines (Fig. 1C).

The PI3K/AKT and RAS signalling pathways are strongly involved in HGSOC, and PTP4A3 has been reported to increase the activity of the PI3K/AKT/mTORC1, MAPK/ERK and/or SRC pathways in distinct cellular types [6, 8, 9, 10]. When the basal activity of these pathways was assessed in Fig. 1D, we found that KRAS amplification in Kuramochi cells [39] correlated with their elevated levels of ERK phosphorylation. OVCAR 4 cells exhibited high PI3K activity, as indicated by phospho‐AKT expression, but lower RAS activity than Kuramochi, in agreement with their KRAS expression status. However, the low KRAS activity in OVCAR 3 cells did not match their elevated KRAS expression, although the lack of PTP4A3 protein expression may partly explain the low activity of both pathways in these cells (Fig. 1D).

Expression of PTP4A3 protein may be controlled through proteostatic mechanisms, with exogenously expressed PTP4A3 behaving as an autophagy substrate [15]. However, when HGSOC cells were treated with or without the inhibitors bafilomycin A1 (BafA1) or MG132, to inhibit autophagy and the ubiquitin‐proteasome systems, respectively, immunoblotting detected no difference in PTP4A3 protein expression levels in any cell line (Fig. 1E).

### 3.2 Amino acid deprivation‐induced autophagy is dysregulated in OVCAR 4 and Kuramochi cells

Given both the paradoxical role of autophagy in cancer and PTP4A3 protein overexpression promoting autophagy to enhance tumour cell growth, the autophagy status of HGSOC cells was examined [15, 19]. To assess basal and inducible autophagy, each cell line was deprived of amino acids (EBSS) for up to 8 h, and the activation of autophagy was quantified by monitoring the conversion of the autophagy marker protein LC3B from LC3B‐I to LC3B‐II. The removal of amino acids results in mTORC1 inhibition concomitant with autophagy activation, which was assessed by monitoring S6K1 Thr389 phosphorylation [41, 42, 43].

**Fig. 2:**
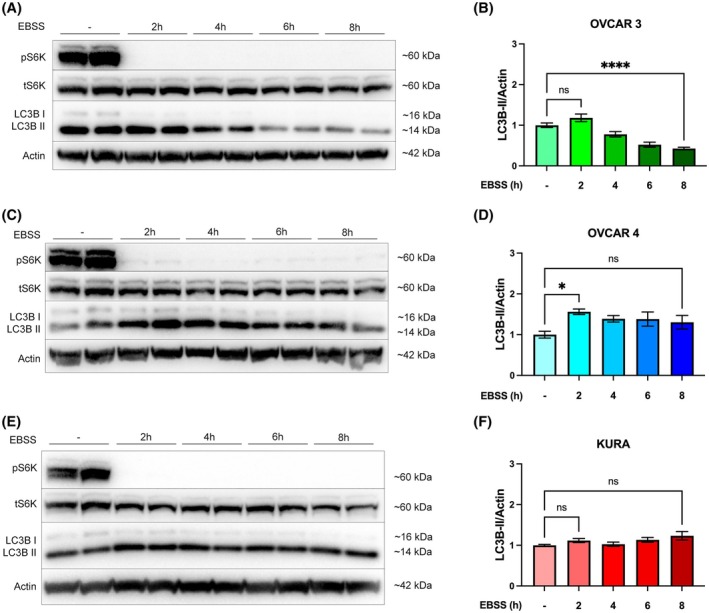
Activation of autophagy by amino acid deprivation is dysregulated in OVCAR 4 and Kuramochi cells. (A) OVCAR 3, (B) OVCAR 4, or (C) Kuramochi cells were incubated with EBSS for 0, 2, 4, 6, and 8 h. The phosphorylation of S6K1 at pThr389 was used to confirm the inhibition of mTORC1 signalling. β‐actin was used as a loading control. (D–F) Autophagy flux was quantified by densitometry of LC3B‐II normalised to β‐actin. Densitometric analysis was pooled from three independent experiments, and error bars = ±SEM. *P < 0.05, ****P < 0.0001 by one‐way ANOVA test.

In all three cell lines, there was robust inhibition of mTORC1 activity after 2 h EBSS treatment (Fig. 2A,C,E). OVCAR 4 cells also significantly induced the formation of LC3B‐II within 2 h EBSS, whereas no statistically significant LC3B‐II induction was observed in OVCAR 3 or Kuramochi cells (compare Fig. 2A,D with Fig. 2B,E and Fig. 2C,F). Despite this, there was a rapid loss of LC3B‐II in OVCAR 3 cells over an extended time frame (Fig. 2A,D), but this was not evident in Kuramochi cells (Fig. 2C,F). Although OVCAR 4 cells rapidly induced LC3B‐II at 2 h following amino acid withdrawal, LC3B‐II turnover was minimal and not statistically significant (Fig. 2B,E). Finally, Kuramochi cells showed minimal activation of autophagy, with no change in LC3B‐II levels following amino acid withdrawal (Fig. 2C,F).

### 3.3 Elevated basal autophagy in OVCAR 3 and Kuramochi, but not OVCAR 4 cells

**Fig. 3:**
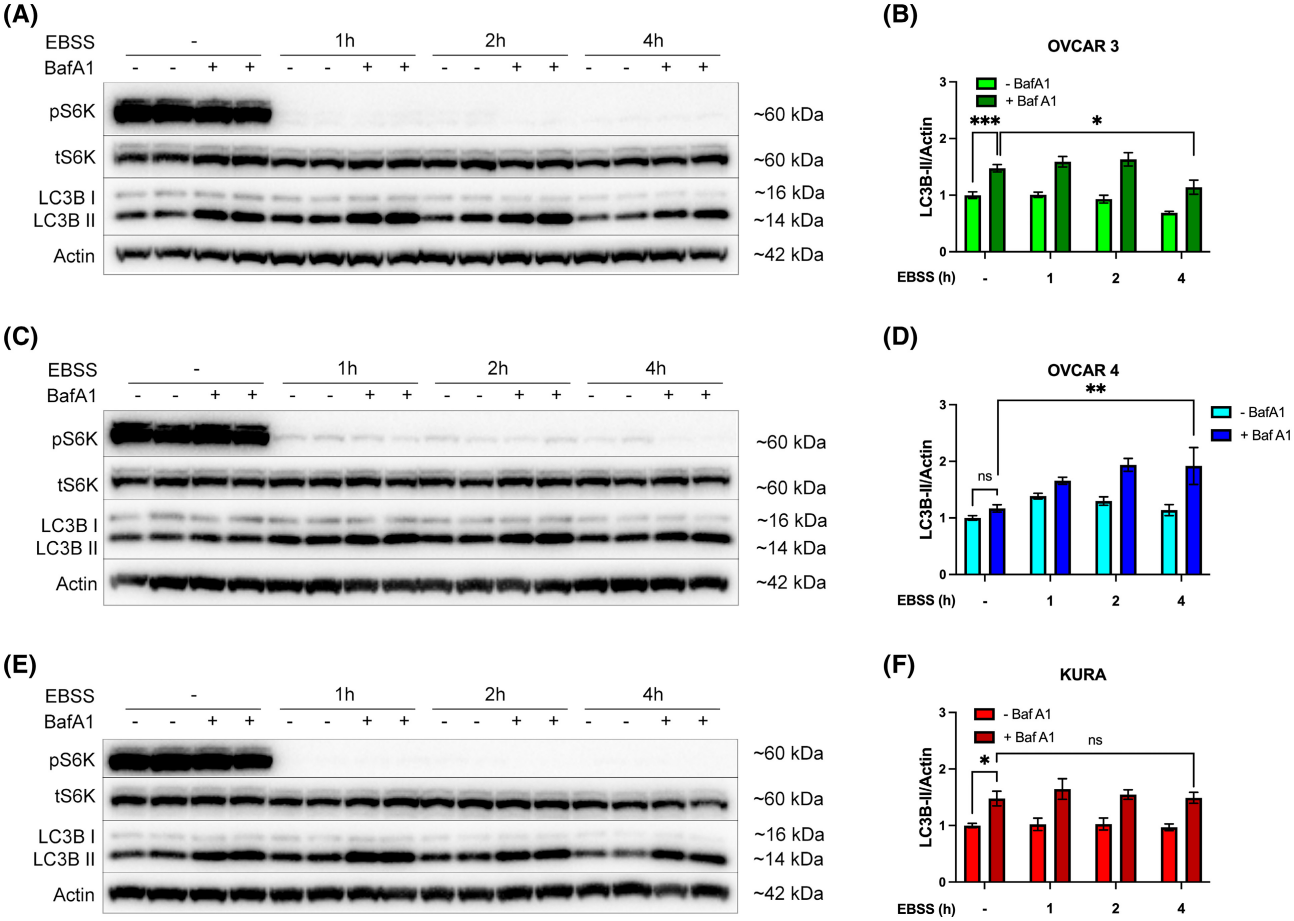
High basal autophagy is detectable in OVCAR3 and Kuramochi, but not OVCAR4 cells. (A) OVCAR 3, (B) OVCAR 4, or (C) Kuramochi cells were incubated with EBSS for 0, 1, 2, or 4 h before the addition of BafA1 (100 nm) for the final hour. The phosphorylation of S6K1 (pThr389) was used to confirm the inhibition of mTORC1 signalling. β‐actin was used as a loading control. (D–F) Autophagy flux was quantified by densitometry of LC3B‐II normalised to β‐actin. Densitometric analysis was pooled from three independent experiments, and error bars = ±SEM. *P < 0.05, **P < 0.01, ***P < 0.001 by two‐way ANOVA test.

Accumulation of autophagosomes can occur following either induction or inhibition of autophagy [23, 44], and this was resolved by measuring autophagy flux using Bafilomycin A1 (BafA1) for the final h of each EBSS timepoint (Fig. 3). OVCAR 3 and Kuramochi cells, but not OVCAR 4 cells, showed statistically significant accumulation of LC3B‐II in nutrient‐replete conditions, indicating a high basal autophagy activity (Fig. 3D,F).

Following amino acid withdrawal, OVCAR 3 cells showed a robust increase in LC3B‐II accumulation after 1 and 2 h EBSS treatment although this decreased by 4 h, indicating time‐dependent attenuation of autophagy (Fig. 3A,D). Conversely, OVCAR 4 cells displayed a much slower accumulation in LC3B‐II up to 4 h, and LC3B‐II levels remained high in the presence of BafA1, indicating that autophagy activity remained elevated even at 4 h (Fig. 3B,E). Finally, in Kuramochi cells, LC3B‐II accumulation in the presence of BafA1 did not increase over time, suggesting that Kuramochi possessed basal but little activatable autophagy, at least in response to amino acid deprivation (Fig. 3C,F).

### 3.4 Basal autophagy in OVCAR 3 and Kuramochi cells is RAS‐dependent

**Fig. 4:**
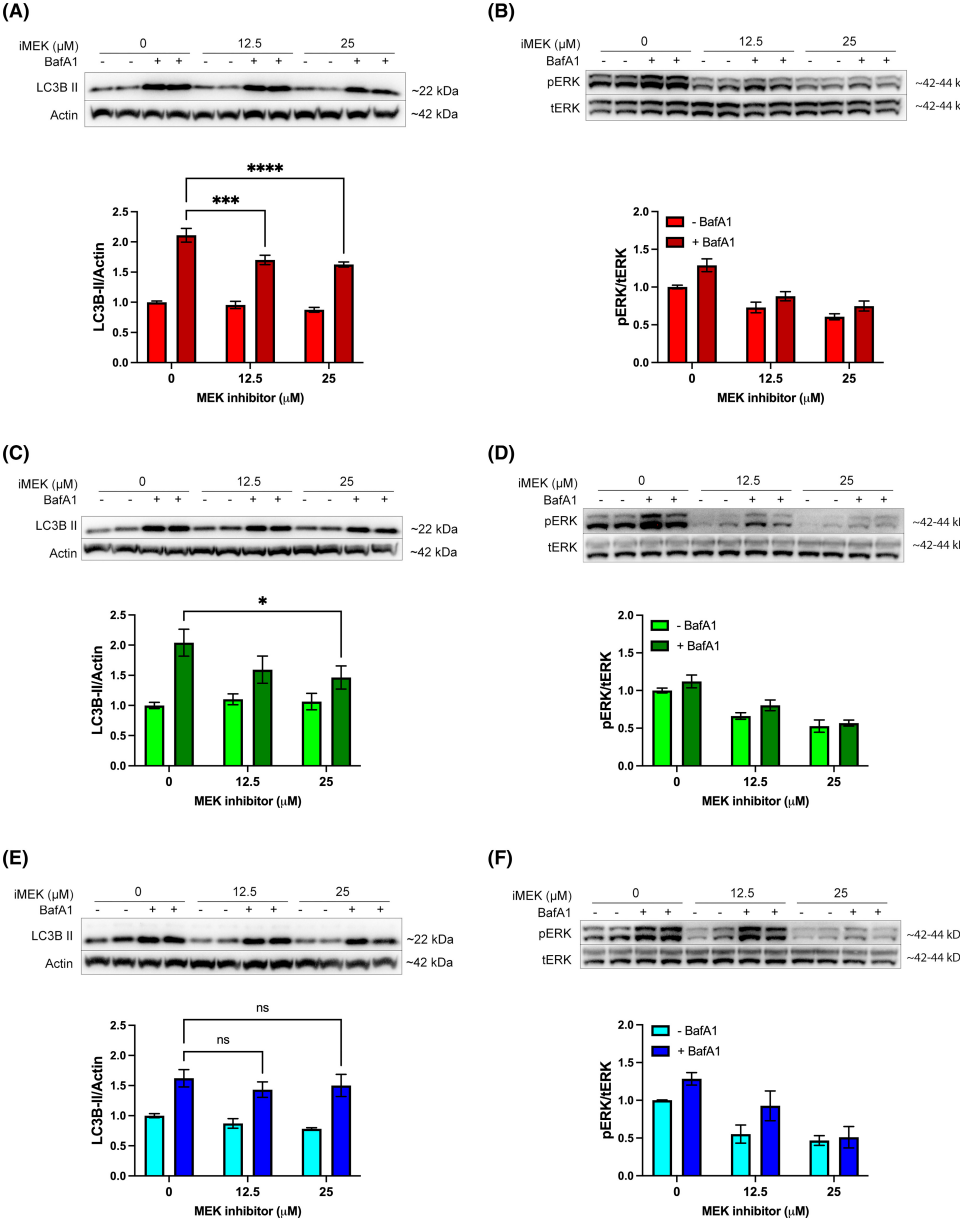
Inhibition of the Ras pathway attenuates high basal autophagy in Kuramochi and OVCAR 3 cells. (A, B) Kuramochi, (C, D) OVCAR 3, and (E, F) OVCAR 4 cells were treated with MEK inhibitor, PD98059 (iMEK; 12.5 or 25 μm) for 24 h in the presence or absence of BafA1 (100 nm) for the last hour. (A, C, E) LC3B was probed as an autophagy marker, and β‐Actin was used as a loading control; autophagy flux was quantified by densitometry of LC3B‐II normalised to β‐actin. (B, D, F) The phosphorylation of ERK1‐2 at pThr202‐Tyr204 was used to confirm RAS pathway inhibition and was quantified by densitometry of phosphorylated ERK normalised to total ERK expression. Densitometric analysis was pooled from a minimum of three independent experiments, and error bars = ±SEM. *P < 0.05, ***P < 0.001, ****P < 0.0001 by two‐way ANOVA test.

Both Kuramochi and OVCAR 3 cells possess high KRAS mRNA expression (Fig. 1C) correlating with elevated basal autophagy (Fig. 3). We hypothesised that this may be another example of RAS‐driven autophagy addiction in cancer [23, 24, 25]. To investigate this, cells were treated with or without a MEK inhibitor to inhibit RAS signalling, and autophagy flux was investigated as before, using BafA1 (Fig. 4). Following inhibition of MEK, ERK1/2 phosphorylation was decreased (Fig. 4B,D,F), as was LC3B‐II accumulation in Kuramochi and OVCAR 3 cells (Fig. 4A,C), but not in OVCAR 4 cells (Fig. 4E). These data establish that RAS‐driven autophagy occurs in Kuramochi and OVCAR 3 cells.

### 3.5 PTP4A3 overexpression in OVCAR 3 cells does not promote autophagy

**Fig. 5:**
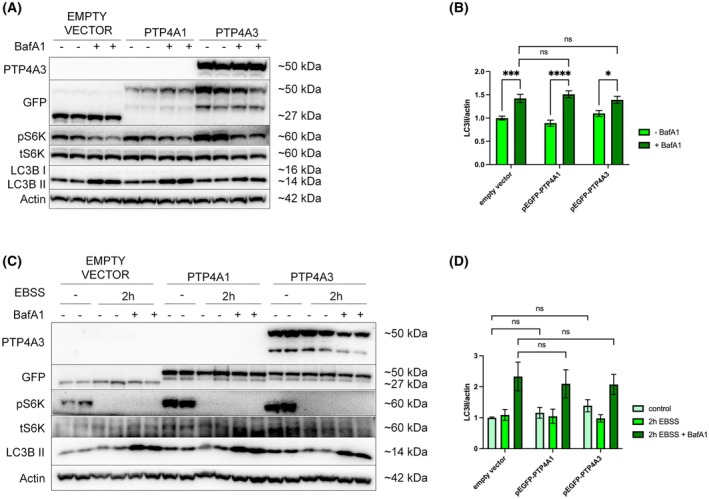
PTP4A3 overexpression in OVCAR 3 cells does not affect either basal or activatable autophagy. OVCAR 3 cells were transiently transfected with either pEGFP (empty vector), pEGFP‐PTP4A1, or pEGFP‐PTP4A3. (A) Cells were incubated with or without 100 nm BafA1 for 1 h. The phosphorylation of S6K1 (pThr389) was used to confirm the inhibition of mTORC1 signalling, and β‐actin was used as a loading control, while GFP was probed as a transfection efficiency control. (B) Basal autophagy activity was quantified by densitometry of LC3B‐II and normalised to β‐actin. (C) Alternatively, cells were incubated with or without EBSS for 2 h in the presence or absence of BafA1 (100 nm) for the last hour. (D) Activatable autophagy was quantified by densitometry of LC3B‐II normalised to β‐actin expression. Data were pooled from three independent experiments, and error bars = ±SEM. *P < 0.05, ***P < 0.001, ****P < 0.0001 by two‐way ANOVA test.

OVCAR 3 cells express negligible PTP4A3 (Fig. 1A–C). Previously, we reported that overexpression of PTP4A3 promoted autophagy in the non‐serous OC cell line, A2780 [15]. To demonstrate if PTP4A3 overexpression also enhances autophagy in OVCAR 3 cells, cells were transiently transfected with wild‐type PTP4A3 (pEGFP‐PTP4A3) or PTP4A1 (pEGFP‐PTP4A1) and an empty vector control (pEGFPC1) (Fig. 5) [15]. Increases in basal autophagy flux were assessed using BafA1. However, no statistically significant increase in LC3B‐II levels was detected in cells overexpressing either PTP4A1 or PTP4A3 (Fig. 5A,B). OVCAR 3 cells overexpressing PTP4A3 were also incubated in EBSS for 2 h with or without BafA1, but again, LC3B‐II levels were no different from either PTP4A1 or empty vector controls (Fig. 5C,D), revealing that PTP4A3 overexpression did not promote autophagy in OVCAR 3 HGSOC cells.

### 3.6 Silencing PTP4A3 expression attenuated autophagy in OVCAR 4 cells

**Fig. 6:**
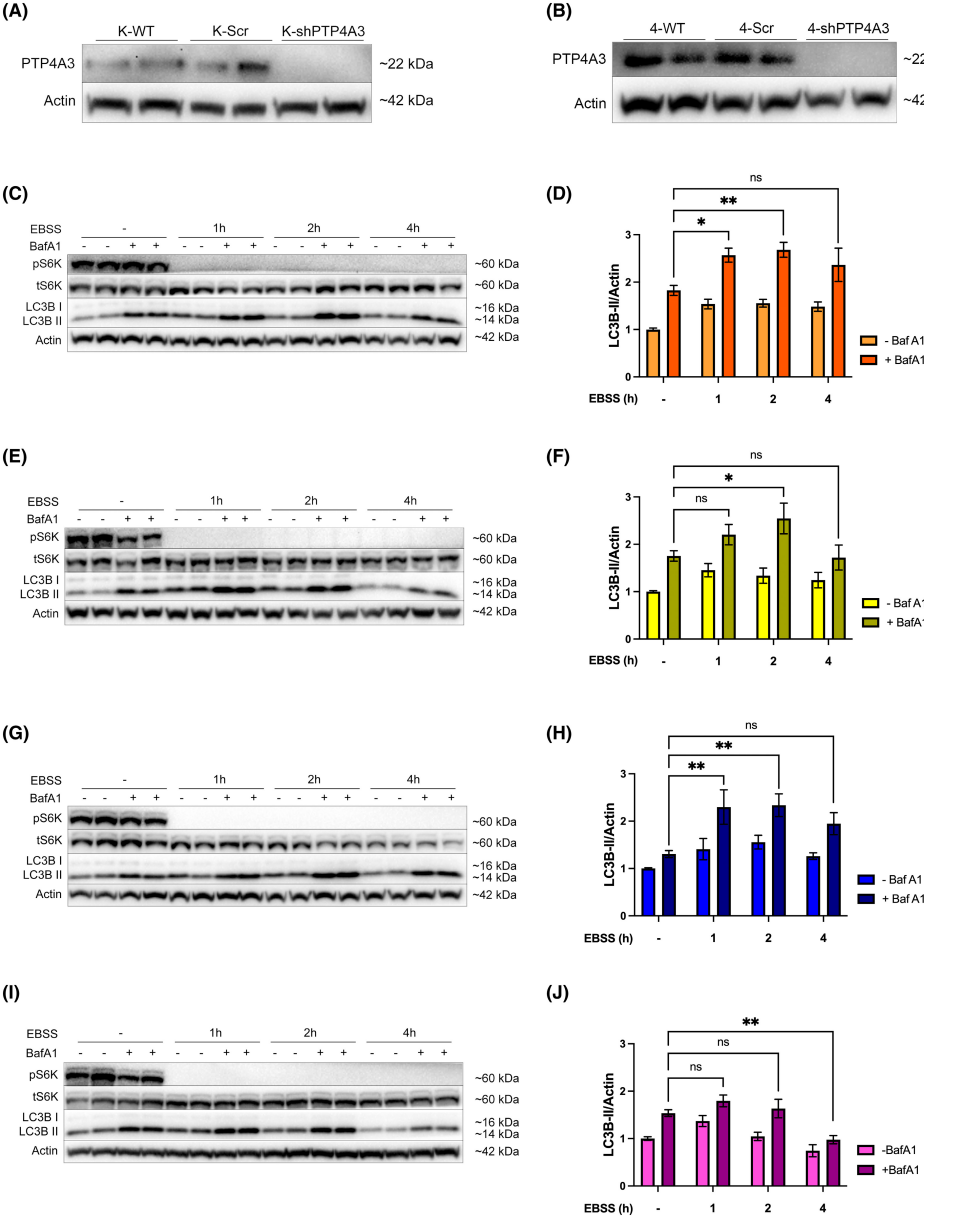
Silencing of PTP4A3 expression attenuated activatable autophagy in OVCAR 4 but not Kuramochi cells. Kuramochi (A) and OVCAR 4 (B) cells were infected with lentiviral particles harbouring either scrambled control shRNA (Scr) or PTP4A3‐targeting shRNA (shPTP4A3). The silencing of PTP4A3 expression was quantified by Western immunoblotting. (C, D) Kuramochi‐Scr (K‐Scr), (E, F) Kuramochi‐shPTP4A3 (K‐shPTP4A3), (G, H) OVCAR 4‐Scr (4‐Scr) and (I, J) OVCAR 4‐shPTP4A3 (4‐shPTP4A3) cells were incubated with EBSS for 0, 1, 2, or 4 h and BafA1 (100 nm) was added for the final hour. (C, E, G, I) The phosphorylation of S6K1 (pThr389) was assessed by western blot to confirm inhibition of mTORC1 signalling. β‐actin was used as a loading control. (D, F, H, J) Activation of autophagy was quantified by densitometry of LC3B‐II normalised to β‐actin. Data were pooled from at least three independent experiments and error bars = ±SEM. *P < 0.05, **P < 0.01 by two‐way ANOVA test.

Since inducible and basal autophagy (Figs 2 and 3) were dysregulated in OVCAR 4 and Kuramochi cells, the impact of PTP4A3 gene silencing on autophagy was assessed using lentivirus‐mediated shRNA in Kuramochi and OVCAR 4 cells (Fig. 6A,B; Fig. S14). In Kuramochi cells, increased LC3B‐II detection was observed following BafA1 treatment under nutrient‐replete conditions in both scrambled control and shPTP4A3 cells (Fig. 6C–F), indicative of high basal autophagy activity. Following amino acid withdrawal combined with BafA1, both control and shPTP4A3‐treated cells showed a significant increase in LC3B‐II accumulation after 1 and 2 h of EBSS treatment (Fig. 6C,D). LC3B‐II accumulation was reduced after 4 h in scrambled and shPTP4A3‐treated cells but was not statistically significant.

In OVCAR 4 scrambled control cells, inducible autophagy shown by LC3B‐II accumulation following 1 and 2 h of amino acid withdrawal was statistically significant (Fig. 6G,H). However, in contrast, in shPTP4A3 OVCAR 4 cells, amino acid withdrawal did not induce autophagy, but instead, there was a significant amount of LC3B‐II turnover at 4 h that did not occur in scrambled control cells (Fig. 6I,J).

### 3.7 Differential PI3K and RAS signalling in Kuramochi and OVCAR 4 cells was revealed by pan‐inhibition of PTP4A1‐3

Studies of PTP4A1 and PTP4A2 have demonstrated that those phosphatases may have overlapping functions with PTP4A3. All three PTP4A phosphatases are involved in regulating cell proliferation, survival, migration and adhesion through p53, Rho‐family GTPase, PI3K, JAK–STAT and RAS pathways, while high expression of each PTP4A is linked to several cancer types [5, 45, 46]. Recently, JMS‐053 (iPRL) has been identified as a reversible and non‐competitive pan‐PTP4A inhibitor that suppresses the migration and viability of OC cells while also preventing the *in vivo* growth of OC xenografts [47, 48].

PTP4A3 is the most frequently overexpressed PTP4A phosphatase in different cancer types, although some cancers also co‐express PTP4A1 and/or PTP4A2 [5, 45]. This suggests that compensatory mechanisms may arise when therapeutically targeting a single PTP4A species in isolation. To understand whether inhibition of all PTP4A proteins altered oncogenic signalling, we analysed the effect of iPRL on cell signalling in the Kuramochi and OVCAR 4 cell lines, with and without PTP4A3 knockdown.

**Fig. 7:**
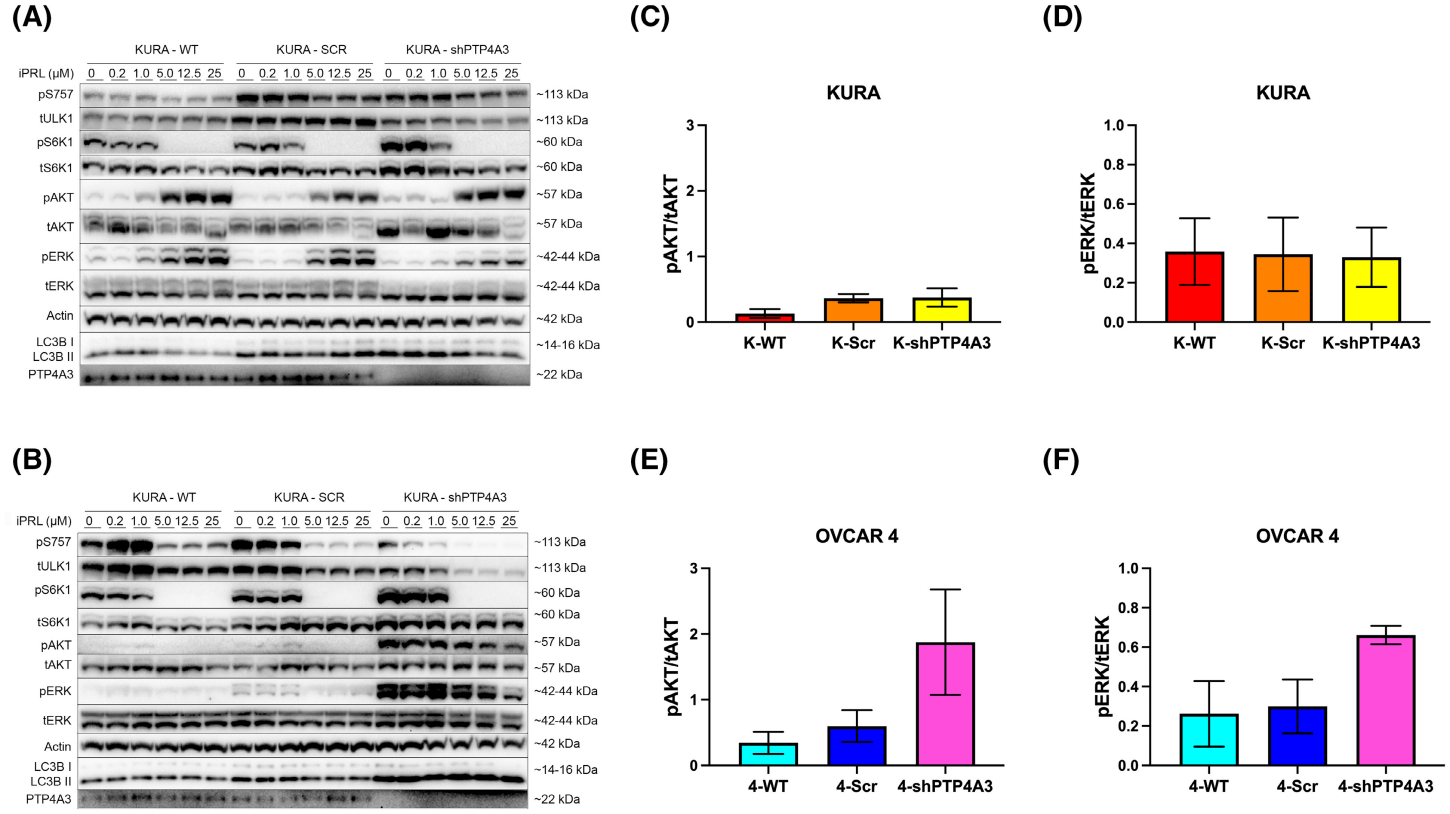
Inhibition of PTP4A1–3 with JMS‐053 revealed differential PI3K and RAS signalling in Kuramochi and OVCAR 4 cells. (A) Kuramochi‐WT, Scr, and shPTP4A3 and (B) OVCAR 4‐WT, Scr and shPTP4A3 cells were treated with increasing concentrations of JMS‐053 (iPRL; 0–25 μm) for 2 h. Cell extracts were analysed for phosphorylation of ULK1 (pSer757), S6K1 (pThr389), AKT/PKB (pSer473) and ERK1‐2 (pThr202‐Tyr204). LC3B was used as a marker of autophagy activity, β‐actin was used as a loading control, and PTP4A3 knockdown was also confirmed. (C–F) The basal phosphorylation of AKT and ERK in WT, Scr, and shPTP4A3 cells (no JMS‐053 treatment) in (C, D) Kuramochi and (E, F) OVCAR 4 cells was determined. Data were pooled from three independent experiments, and error bars = ±SEM.

Incubation of Kuramochi cells with ≥5 μm iPRL activated PI3K and RAS signalling, detected by increased AKT and ERK1/2 phosphorylation (Fig. 7A). In contrast, iPRL inhibited PI3K and RAS signalling in OVCAR 4 cells (Fig. 7B). Strikingly, ≥5 μm iPRL inhibited S6K1 phosphorylation in WT, scrambled control and shPTP4A3 knockdowns of both cell lines (Fig. 7A,B), which was associated with decreased ULK1 phosphorylation at Ser 757, the mTORC1 inhibitory site, suggesting that targeting pan‐PTP4As inhibited mTORC1 at a point distal to AKT signalling.

Distinct differences in PI3K and RAS signalling were observed in OVCAR 4 cells following PTP4A3 silencing (Fig. 7B). Quantification of AKT and ERK1/2 phosphorylation revealed no change in Kuramochi shPTP4A3 cells compared to control cells (Fig. 7C,D). Conversely, silencing PTP4A3 in OVCAR 4 cells elevated both AKT (Ser473) and ERK1/2 phosphorylation, although these effects were not statistically significant (Fig. 7E,F).

### 3.8 PTP4A3 knockdown sensitises K‐Ras mutant HGSOC to 5‐fluorouracil

Most women with advanced OC initially respond favourably to first‐line treatments, carboplatin and paclitaxel. However, patients develop chemoresistance leading to treatment failure in 80–90% of cases, with a 5‐year survival rate of <40% for women with HGSOC [49, 50, 51, 52]. OC heterogeneity represents a major challenge for the improvement of cure rates for this disease [49, 51]. The HGSOC cell lines used in this study display different levels of PTP4A3 and KRAS expression. To translate our results towards improved therapeutic strategies, we assessed whether PTP4A3 knockdown sensitised HGSOC cells towards pan‐PTP4A inhibition (iPRL) or to the clinically relevant chemotherapeutic agents, fluorouracil (5FU), cisplatin (CDDP) and paclitaxel (PTX). Although 5‐FU is not currently a standard‐of‐care therapy for ovarian cancer, the standard treatments for this malignancy often lead to the development of chemoresistance. It is therefore essential to explore the potential efficacy of alternative chemotherapeutic agents. Furthermore, the combination of PTP4A3 inhibition/knockdown with these chemotherapeutic agents represents a novel approach that has not previously been tested in HGSOC cells. The use of 5‐FU provides a valuable comparison between standard‐of‐care therapies and alternative treatments in this experimental setting, offering broader insights into potential therapeutic strategies.

We employed time‐lapse cell culture imaging and analysis to quantify cell confluency and incorporation of propidium iodide (PI), as measures of reduced cell proliferation and cell death, respectively. We found that Kuramochi cells may be more dependent on PTP4A3 activity given their higher PTP4A3 expression, which resulted in greater sensitivity towards iPRL compared to OVCAR 4 and OVCAR 3 (Fig. S2), while PTP4A3 silencing in both Kuramochi and OVCAR 4 cell lines also appeared to sensitise these cells to iPRL (Figs S3 and S4). Moreover, the absence of PTP4A3 expression was associated with greater sensitivity to chemotherapeutic drugs. OVCAR 3 cells were more sensitive to CDDP and PTX than WT Kuramochi or OVCAR 4 cells (Figs S8 and S11), whereas Kuramochi were more sensitive to 5‐FU than OVCAR 3 and OVCAR 4 cells (Fig. S5). The shPTP4A3 Kuramochi cell line was more sensitive to 5‐FU, CDDP and PTX than their control counterparts (Figs S6, S9, S12), while knocking down PTP4A3 in OVCAR 4 cells sensitised them towards 5‐FU and PTX (Figs S7 and S13), but not CDDP (Fig. 10).

**Fig. 8:**
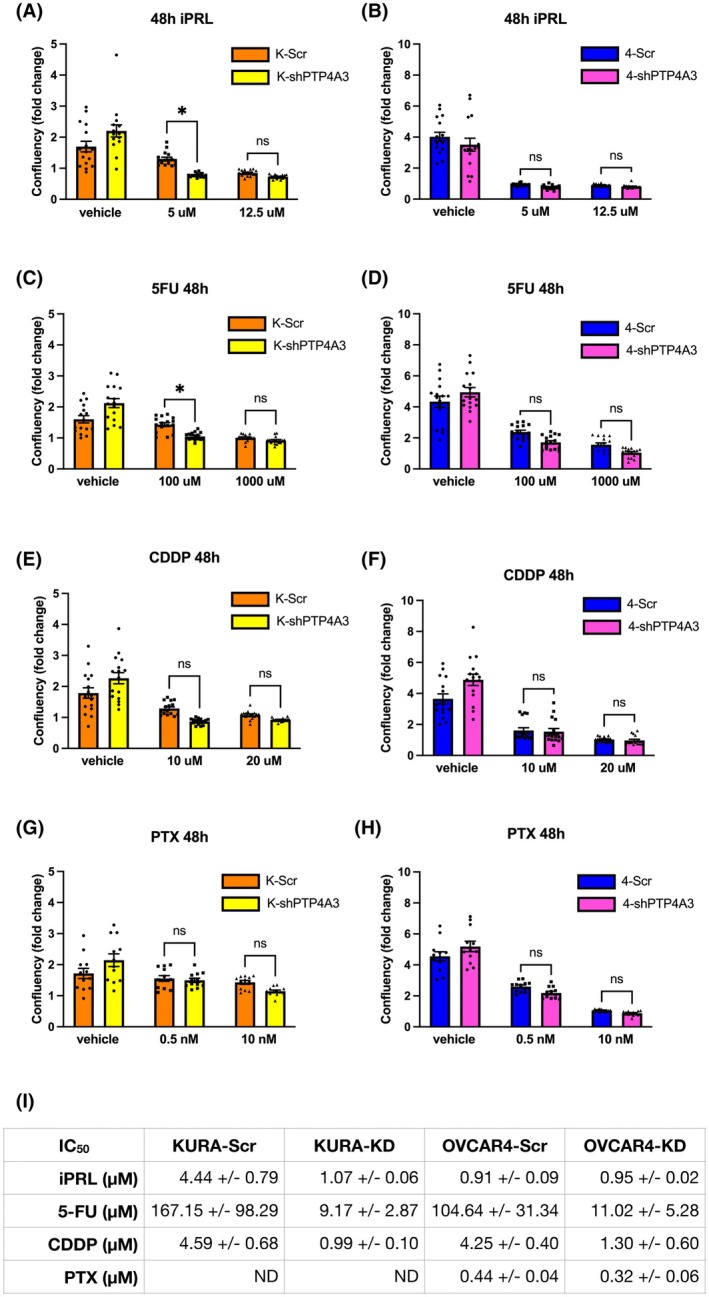
Silencing of PTP4A3 sensitises K‐Ras mutant, but not K‐Ras wild‐type HGSOC to iPRL and 5FU. K‐Scr, K‐shPTP4A3, OVCAR 4‐Scr, and OVCAR 4‐shPTP4A3 cells were treated with; (A, B) JMS‐053 (iPRL), (C, D) 5‐fluorouracil (5FU), (E, F) cisplatin (CDDP) or (G, H) paclitaxel (PTX) for 72 h in an Incucyte S3 system. (A–H) Cell confluency at 48 h relative to T_0_. Data was pooled from three (PTX) or four (iPRL, 5FU and CDDP) independent experiments and error bars = ±SEM, with *P < 0.05 by two‐way ANOVA test. (I) Table summarising the IC_50_ values for each drug ± SEM.

Compared to scrambled control, shPTP4A3 Kuramochi cells treated with 5 μm iPRL had significantly lower cell confluency at 48 h (Fig. 8A). Similarly, silencing PTP4A3 in Kuramochi cells sensitised them towards 100 μm 5‐FU (Fig. 8C). We also observed a trend of increased sensitivity to 10 μm CDDP (Fig. 8E) and 10 nm PTX (Fig. 8G) in Kuramochi PTP4A3 knockdown cells. However, silencing PTP4A3 in OVCAR 4 cells did not alter drug sensitivity compared to scrambled controls (Fig. 8B,D,F,H). Nonetheless, the calculated IC_50_ values for each drug revealed increased sensitivity towards 5‐FU and CDDP following PTP4A3 silencing in Kuramochi and OVCAR 4 cells (Fig. 8I; Fig. S1). We also noted that iPRL‐treated shPTP4A3 Kuramochi cells had a 4‐fold lower IC_50_ compared to scrambled control (Fig. 8I; Figs S1, S3), suggesting that compensatory mechanisms occur between PTP4A proteins.

## 4 Discussion

PTP4A3 is overexpressed in 80.6% of randomly analysed human tumours of diverse cancer types, while absent in all examined normal tissues. This makes it a highly promising tumour antigen for the development of cancer therapies. For example, PRL3‐zumab, a first‐in‐class humanised mAb in phase 1 clinical trials, has been reported to promote the infiltration of B cells, NK cells and macrophages into the tumour microenvironment, leading to a ‘Kill‐and‐leak’ cascade [12, 13]. Numerous studies have reported the involvement of PTP4A3 in malignant transformation and EMT through several oncogenic pathways, including the PI3K/AKT/mTORC1, RAS and SRC signalling pathways [30, 53]. Further, we previously demonstrated the ability of PTP4A3 overexpression to promote autophagy in A2780 cells [15]. Thus, we sought to delineate the molecular mechanisms by which PTP4A3 mediates cell growth, proliferation and autophagy in a panel of HGSOC cells.

Analysis of RNA and protein expression revealed that PTP4A3 expression levels varied across the three HGSOC cell lines examined. We report that Kuramochi cells had the highest levels of PTP4A3 mRNA and protein expression and ERK activity, as compared to the OVCAR‐3 and OVCAR‐4 cell lines. Kuramochi cells have been previously reported to harbour KRAS amplification, which is highly effective at driving RAS signalling, contributing to proliferation, differentiation and growth [39, 54, 55]. Thus, the significantly elevated ERK phosphorylation levels detected in Kuramochi cells are likely due to the combination of their high PTP4A3 protein expression and mutant KRAS status. The over‐activated RAS pathway in Kuramochi cells may also result in the observed low PI3K signalling activity due to known pathway crosstalk [56]. Elevated basal and minimal activatable autophagy is a common phenotype of KRAS‐driven, autophagy‐addicted cancers [22, 23, 24, 25]. Consistent with this phenotype, amino acid deprivation combined with autophagy flux analysis (using BafA1) revealed that Kuramochi cells displayed high basal but no activatable autophagy activity (Figs 2 and 3).

Conversely, PTP4A3 protein expression in the absence of KRAS amplification was shown in OVCAR 4 cells, which may be the reason why this cell line showed high PI3K and some RAS activity (although lower than Kuramochi cells). We also observed that OVCAR 4 cells had low basal and low activatable autophagy profiles. In contrast, OVCAR 3 cells did not express PTP4A3 mRNA and protein, and while they exhibited elevated KRAS mRNA expression, it was still below the levels of Kuramochi cells. These two features may, therefore, explain the low RAS and PI3K pathway activities that were observed for the OVCAR 3 cell line, compared to Kuramochi and OVCAR 4 cells. However, OVCAR 3 cells were shown to possess high basal and activatable autophagy levels (Figs 2 and 3). Inhibition of MEK signalling was able to reduce basal autophagy in both OVCAR 3 and Kuramochi cells but not in OVCAR 4 cells, strongly suggesting that basal autophagy in Kuramochi and OVCAR 3 cells is KRAS‐driven, whereas autophagy in OVCAR 4 cells is independent of RAS signalling (Fig. 4).

Further investigation into the importance of PTP4A3 in the regulation of autophagy in HGSOC cells revealed that transient transfection of PTP4A3 protein in OVCAR 3 cells did not affect basal or activatable autophagy (Fig. 5). This result is in contrast with our previous findings in A2780 cells. However, these cells differ genetically from OVCAR 3 cells as they do not harbour any KRAS mutation or amplification [15]. Our findings, therefore, support the hypothesis that KRAS‐driven autophagy in OVCAR 3 cells may mask any effects of PTP4A3 overexpression on autophagy. In this regard, Fig. 6 provides further support for this hypothesis, showing that PTP4A3 silencing in Kuramochi cells did not alter autophagy, while in OVCAR 4 cells (which do not express KRAS), PTP4A3 silencing led to decreased activatable autophagy. Therefore, endogenous PTP4A3 expression appears to be necessary and sufficient for autophagy induction in KRAS‐negative HGSOC cells (such as OVCAR 4 and A2780) whereas KRAS drives autophagy when expressed in HGSOC cells (such as Kuramochi and OVCAR 3), overriding any effects of PTP4A3 on autophagy.

This study also addressed whether potential compensatory mechanisms occurred between PTP4A1–3 by employing a pan‐PRL inhibitor (iPRL). While the iPRL inhibitor increased PI3K and RAS signalling in Kuramochi cells, it had the opposite effect in OVCAR 4 cells, decreasing PI3K and RAS signalling and did not alter PTP4A3 protein expression (Fig. 7). Additionally, iPRL treatment led to attenuated mTORC1‐dependent S6K1 and also ULK1 phosphorylation in all cell lines tested. This is in line with previous studies, which showed that PTP4A3 overexpression induces increased phosphorylation of mTORC1, 4E‐BP1 and p70S6K [57] and that PTP4A3 is involved in the regulation of PI3K signalling with reduced levels of AKT and mTOR phosphorylation in PTP4A3 knockdown glioblastoma LN229 and U87 cell lines [58]. Moreover, PTP4A3 knockdown cell lines showed a similar response to iPRL compared to control cells. Given that these cells also express PTP4A1 and/or PTP4A2, shown by RNA‐seq analysis (Fig. 1C) and that all three PTP4A phosphatases can participate in the regulation of RAS and PI3K signalling [5, 45], then targeting only PTP4A3 via shRNA silencing likely resulted in the upregulation of compensatory PTP4A1 and PTP4A2 mechanisms (Fig. 7E,F). Conversely, since high KRAS expression appears to alter these signalling pathways in Kuramochi, silencing of PTP4A3 did not elicit these effects in this HGSOC line (Fig. 7C,D). Pan‐PTP4A1–3 inhibition in Kuramochi was only efficient at inhibiting mTORC1 activity, suggesting that PTP4A1‐3s may function at different points along the PI3K and RAS pathways, including proximal to mTORC1, although high KRAS expression did not impact mTORC1 activity.

Finally, we considered the reported roles of PTP4A3 in tumour recurrence and chemotherapy resistance, where its mRNA and protein expression are upregulated following exposure to chemotherapeutic drugs [59, 60]. Assays of cell proliferation and death revealed that the highest expression of PTP4A3, found in Kuramochi cells, also correlated with the greatest sensitivity to iPRL (Fig. S2), suggesting that the viability of cells with higher PTP4A3 protein expression is more dependent on the activity of this phosphatase. Additionally, the absence of PTP4A3 in OVCAR 3 cells corresponded to increased CDDP and PTX cytotoxicity (Figs S8, S11) when compared to Kuramochi and OVCAR 4 cells, in line with the hypothesis of PTP4A3 having a role in chemoresistance. A statistically significant sensitisation was observed in PTP4A3‐silenced Kuramochi cells to iPRL and 5FU (Fig. 8A,C). In addition, trends towards increased sensitivity of PTP4A3‐silenced Kuramochi cells to 5‐FU, CDDP and PTX (Figs S6, S9, S12), and PTP4A3‐silenced OVCAR 4 cells to 5‐FU and PTX (Figs S7 and S13) were also observed, compared to scramble controls. The IC_50_ values for 5FU and CDDP were ≥4 times lower in PTP4A3‐silenced OVCAR 4 cells, and also for 5FU, CDDP and iPRL in PTP4A3‐silenced Kuramochi cells. These results suggest that targeting PTP4A3 expression in HGSOC sensitises them to DNA‐damaging chemotherapeutics, and this approach is also more effective in KRAS mutant HGSOC cells.

## 5 Conclusions

This study has demonstrated that compensatory mechanisms from PTP4A1 and PTP4A2 can arise when specifically targeting PTP4A3 in HGSOC and that pan‐PTP4A inhibition can overcome those effects. Moreover, KRAS‐driven tumours may compensate for the lack of PTP4A1–3 activity by increasing RAS and PI3K signalling, coupled with autophagy addiction. Furthermore, the autophagy‐promoting effects of PTP4A3 are dependent on KRAS status in OC, and having a KRAS dominant activity generates an autophagy addiction that cannot be altered by modulating PTP4A3 expression. Importantly, however, a combinatorial approach of targeting PTP4A3 with PRL3‐zumab, which has already been shown effective in mice and currently is in Phase II trials (NCT04452955), alongside current generation, clinically used chemotherapeutic drugs (5‐fluorouracil, cisplatin and paclitaxel) could be an effective strategy for treating KRAS mutant, HGSOC cancers.

## Conflict of interest

The authors report no competing interests.

## Author contributions

ALG contributed to the conceptualisation of experiments, the generation of *in vitro* data, and the analysis and interpretation of results. ALG also wrote the manuscript. DJ was responsible for generating the normalised RNA‐seq data and its analysis. EC was responsible for the analysis of experimental data, interpreting results, and writing and providing feedback on the manuscript. JTM was responsible for conceptualising the experimental approach, generation and analysis of experimental data, interpreting the results, and writing and providing feedback on the manuscript.

## Peer review

The peer review history for this article is available at https://www.webofscience.com/api/gateway/wos/peer‐review/10.1002/1878‐0261.70092.

## Supporting information


**Fig. S1.** IC50 curves for JMS‐053, 5FU, CDDP and PTX.
**Fig. S2.** Kuramochi cells show higher sensitivity to the pan‐PTP4A/PRL inhibitor (iPRL) than OVCAR 3 and OVCAR 4 cells.
**Fig. S3.** Kuramochi‐KD (K‐KD) cells show higher sensitivity to PRL inhibitor (iPRL) than K‐Scr cells, however, K‐Scr shows higher resistance than K‐WT cells.
**Fig. S4.** OVCAR 4‐KD (4‐KD) cells show higher sensitivity to the PRL inhibitor (iPRL) than 4‐WT and 4‐Scr cells.
**Fig. S5.** Kuramochi cells show higher sensitivity to 5FU than OVCAR 3 and OVCAR 4.
**Fig. S6.** Kuramochi‐KD (K‐KD) cells show higher sensitivity to 5FU than K‐Scr, however, K‐Scr shows higher resistance than K‐WT.
**Fig. S7.** OVCAR 4‐KD (4‐KD) cells show higher sensitivity to 5FU than 4‐WT and 4‐Scr.
**Fig. S8.** OVCAR 3 cells show higher sensitivity to cisplatin (CDDP) than OVCAR 4 and Kuramochi.
**Fig. S9.** Kuramochi‐KD (K‐KD) cells show higher sensitivity to cisplatin (CDDP) than K‐WT and K‐Scr.
**Fig. S10.** *PTP4A3* silencing does not produce a significant effect in the response of OVCAR 4 cells to cisplatin (CDDP) treatment.
**Fig. S11.** OVCAR 3 cells show higher sensitivity to paclitaxel (PTX) than OVCAR 4 and Kuramochi.
**Fig. S12.** Kuramochi‐KD (K‐KD) cells show higher sensitivity to paclitaxel (PTX) than K‐Scr, however, K‐WT is the most sensitive.
**Fig. S13.** OVCAR 4‐KD (4‐KD) cells show higher sensitivity to paclitaxel (PTX) than 4‐WT and 4‐Scr.
**Fig. S14.** PTP4A3 mRNA expression in OVCAR 4 and Kuramochi cells upon lentiviral‐mediated shRNA knockdown.

## Data Availability

All relevant materials and reagents are freely available upon request from the corresponding author, as are all raw data to any researcher wishing to use them for non‐commercial purposes.

## References

[1] Kroeger PT Jr , Drapkin R . Pathogenesis and heterogeneity of ovarian cancer. Curr Opin Obstet Gynecol. 2017;29(1):26–34. 27898521 10.1097/GCO.0000000000000340 PMC5201412

[2] Momenimovahed Z , Tiznobaik A , Taheri S , Salehiniya H . Ovarian cancer in the world: epidemiology and risk factors. Int J Womens Health. 2019;11:287–299. 31118829 10.2147/IJWH.S197604 PMC6500433

[3] Bray F , Ferlay J , Soerjomataram I , Siegel RL , Torre LA , Jemal A . Global cancer statistics 2018: GLOBOCAN estimates of incidence and mortality worldwide for 36 cancers in 185 countries. CA Cancer J Clin. 2018;68(6):394–424. 30207593 10.3322/caac.21492

[4] Zeng Q , Hong W , Tan YH . Mouse PRL‐2 and PRL‐3, two potentially prenylated protein tyrosine phosphatases homologous to PRL‐1. Biochem Biophys Res Commun. 1998;244(2):421–427. 9514946 10.1006/bbrc.1998.8291

[5] Hardy S , Kostantin E , Hatzihristidis T , Zolotarov Y , Uetani N , Tremblay ML . Physiological and oncogenic roles of the PRL phosphatases. FEBS J. 2018;285(21):3886–3908. 29770564 10.1111/febs.14503

[6] Guzinska‐Ustymowicz K , Pryczynicz A . PRL‐3, an emerging marker of carcinogenesis, is strongly associated with poor prognosis. Anticancer Agents Med Chem. 2011;11(1):99–108. 21291404 10.2174/187152011794941145

[7] Bessette DC , Qiu D , Pallen CJ . PRL PTPs: mediators and markers of cancer progression. Cancer Metastasis Rev. 2008;27(2):231–252. 18224294 10.1007/s10555-008-9121-3

[8] Peng L , Xing X , Li W , Qu L , Meng L , Lian S , et al. PRL‐3 promotes the motility, invasion, and metastasis of LoVo colon cancer cells through PRL‐3‐integrin beta1‐ERK1/2 and‐MMP2 signaling. Mol Cancer. 2009;8:110. 19930715 10.1186/1476-4598-8-110 PMC2792223

[9] Al‐Aidaroos AQ , Yuen HF , Guo K , Zhang SD , Chung TH , Chng WJ , et al. Metastasis‐associated PRL‐3 induces EGFR activation and addiction in cancer cells. J Clin Invest. 2013;123(8):3459–3471. 23867504 10.1172/JCI66824 PMC4011027

[10] Rubio T , Kohn M . Regulatory mechanisms of phosphatase of regenerating liver (PRL)‐3. Biochem Soc Trans. 2016;44(5):1305–1312. 27911713 10.1042/BST20160146 PMC5095905

[11] Ye Z , Ng CP , Liu H , Bao Q , Xu S , Zu D , et al. PRL1 and PRL3 promote macropinocytosis via its lipid phosphatase activity. Theranostics. 2024;14(9):3423–3438. 38948056 10.7150/thno.93127 PMC11209707

[12] Thura M , al‐Aidaroos AQO , Yong WP , Kono K , Gupta A , Lin YB , et al. PRL3‐zumab, a first‐in‐class humanized antibody for cancer therapy. JCI Insight. 2016;1(9):e87607. 27699276 10.1172/jci.insight.87607 PMC5033845

[13] Thura M , al‐Aidaroos AQ , Gupta A , Chee CE , Lee SC , Hui KM , et al. PRL3‐zumab as an immunotherapy to inhibit tumors expressing PRL3 oncoprotein. Nat Commun. 2019;10(1):2484. 31171773 10.1038/s41467-019-10127-x PMC6554295

[14] Bersini S , Arrojo E Drigo R , Huang L , Shokhirev MN , Hetzer MW . Transcriptional and functional changes of the human microvasculature during physiological aging and Alzheimer disease. Adv Biosyst. 2020;4(5):e2000044. 32402127 10.1002/adbi.202000044

[15] Huang YH , al‐aidaroos AQO , Yuen HF , Zhang SD , Shen HM , Rozycka E , et al. A role of autophagy in PTP4A3‐driven cancer progression. Autophagy. 2014;10(10):1787–1800. 25136802 10.4161/auto.29989 PMC4198363

[16] Ma Y , Galluzzi L , Zitvogel L , Kroemer G . Autophagy and cellular immune responses. Immunity. 2013;39(2):211–227. 23973220 10.1016/j.immuni.2013.07.017

[17] Ozpolat B , Benbrook DM . Targeting autophagy in cancer management – strategies and developments. Cancer Manag Res. 2015;7:291–299. 26392787 10.2147/CMAR.S34859 PMC4573074

[18] Glick D , Barth S , Macleod KF . Autophagy: cellular and molecular mechanisms. J Pathol. 2010;221(1):3–12. 20225336 10.1002/path.2697 PMC2990190

[19] Onorati AV , Dyczynski M , Ojha R , Amaravadi RK . Targeting autophagy in cancer. Cancer. 2018;124(16):3307–3318. 29671878 10.1002/cncr.31335 PMC6108917

[20] Zhan L , Zhang Y , Wang W , Song E , Fan Y , Li J , et al. Autophagy as an emerging therapy target for ovarian carcinoma. Oncotarget. 2016;7(50):83476–83487. 27825125 10.18632/oncotarget.13080 PMC5347782

[21] Poillet‐Perez L , Despouy G , Delage‐Mourroux R , Boyer‐Guittaut M . Interplay between ROS and autophagy in cancer cells, from tumor initiation to cancer therapy. Redox Biol. 2015;4:184–192. 25590798 10.1016/j.redox.2014.12.003 PMC4803791

[22] Mancias JD , Kimmelman AC . Targeting autophagy addiction in cancer. Oncotarget. 2011;2(12):1302–1306. 22185891 10.18632/oncotarget.384 PMC3282086

[23] Guo JY , Chen HY , Mathew R , Fan J , Strohecker AM , Karsli‐Uzunbas G , et al. Activated Ras requires autophagy to maintain oxidative metabolism and tumorigenesis. Genes Dev. 2011;25(5):460–470. 21317241 10.1101/gad.2016311 PMC3049287

[24] Kim MJ , Woo SJ , Yoon CH , Lee JS , An S , Choi YH , et al. Involvement of autophagy in oncogenic K‐Ras‐induced malignant cell transformation. J Biol Chem. 2011;286(15):12924–12932. 21300795 10.1074/jbc.M110.138958 PMC3075639

[25] Lock R , Roy S , Kenific CM , Su JS , Salas E , Ronen SM , et al. Autophagy facilitates glycolysis during Ras‐mediated oncogenic transformation. Mol Biol Cell. 2011;22(2):165–178. 21119005 10.1091/mbc.E10-06-0500 PMC3020913

[26] Mizushima N , Komatsu M . Autophagy: renovation of cells and tissues. Cell. 2011;147(4):728–741. 22078875 10.1016/j.cell.2011.10.026

[27] Peracchio C , Alabiso O , Valente G , Isidoro C . Involvement of autophagy in ovarian cancer: a working hypothesis. J Ovarian Res. 2012;5(1):22. 22974323 10.1186/1757-2215-5-22 PMC3506510

[28] White E . Deconvoluting the context‐dependent role for autophagy in cancer. Nat Rev Cancer. 2012;12(6):401–410. 22534666 10.1038/nrc3262 PMC3664381

[29] Rouleau C , Roy A , St. Martin T , Dufault MR , Boutin P , Liu D , et al. Protein tyrosine phosphatase PRL‐3 in malignant cells and endothelial cells: expression and function. Mol Cancer Ther. 2006;5(2):219–229. 16505094 10.1158/1535-7163.MCT-05-0289

[30] Wang H , Quah SY , Dong JM , Manser E , Tang JP , Zeng Q . PRL‐3 down‐regulates PTEN expression and signals through PI3K to promote epithelial‐mesenchymal transition. Cancer Res. 2007;67(7):2922–2926. 17409395 10.1158/0008-5472.CAN-06-3598

[31] Reich R , Hadar S , Davidson B . Expression and clinical role of protein of regenerating liver (PRL) phosphatases in ovarian carcinoma. Int J Mol Sci. 2011;12(2):1133–1145. 21541048 10.3390/ijms12021133 PMC3083695

[32] Cell line OVCAR‐3 (CVCL_0465). SIB Swiss Institute of Bioinformatics. https://web.expasy.org/cellosaurus/CVCL_0465

[33] Hamilton TC , Young RC , McKoy W , Grotzinger KR , Green JA , Chu EW , et al. Characterization of a human ovarian carcinoma cell line (NIH:OVCAR‐3) with androgen and estrogen receptors. Cancer Res. 1983;43(11):5379–5389. 6604576

[34] Cell line OVCAR‐4 (CVCL_1627). SIB Swiss Institute of Bioinformatics. https://web.expasy.org/cellosaurus/CVCL_1627

[35] Cell line Kuramochi (CVCL_1345). SIB Swiss Institute of Bioinformatics. https://web.expasy.org/cellosaurus/CVCL_1345

[36] KURAMOCHI. EMBL‐EBI. https://www.ebi.ac.uk/ols/ontologies/efo/terms?short_form=EFO_0006625

[37] Murray JT , Campbell DG , Morrice N , Auld GC , Shpiro N , Marquez R , et al. Exploitation of KESTREL to identify NDRG family members as physiological substrates for SGK1 and GSK3. Biochem J. 2004;384(Pt 3):477–488. 15461589 10.1042/BJ20041057 PMC1134133

[38] Domcke S , Sinha R , Levine DA , Sander C , Schultz N . Evaluating cell lines as tumour models by comparison of genomic profiles. Nat Commun. 2013;4:2126. 23839242 10.1038/ncomms3126 PMC3715866

[39] Harmonizome.

[40] Basak S , Jacobs SBR , Krieg AJ , Pathak N , Zeng Q , Kaldis P , et al. The metastasis‐associated gene Prl‐3 is a p53 target involved in cell‐cycle regulation. Mol Cell. 2008;30(3):303–314. 18471976 10.1016/j.molcel.2008.04.002 PMC3951836

[41] Kim J , Kundu M , Viollet B , Guan KL . AMPK and mTOR regulate autophagy through direct phosphorylation of Ulk1. Nat Cell Biol. 2011;13(2):132–141. 21258367 10.1038/ncb2152 PMC3987946

[42] Populo H , Lopes JM , Soares P . The mTOR signalling pathway in human cancer. Int J Mol Sci. 2012;13(2):1886–1918. 22408430 10.3390/ijms13021886 PMC3291999

[43] Britten CD . PI3K and MEK inhibitor combinations: examining the evidence in selected tumor types. Cancer Chemother Pharmacol. 2013;71(6):1395–1409. 23443307 10.1007/s00280-013-2121-1

[44] Kocaturk NM , Akkoc Y , Kig C , Bayraktar O , Gozuacik D , Kutlu O . Autophagy as a molecular target for cancer treatment. Eur J Pharm Sci. 2019;134:116–137. 30981885 10.1016/j.ejps.2019.04.011

[45] Wei M , Korotkov KV , Blackburn JS . Targeting phosphatases of regenerating liver (PRLs) in cancer. Pharmacol Ther. 2018;190:128–138. 29859177 10.1016/j.pharmthera.2018.05.014 PMC6192704

[46] Hu H , Ye L , Liu Z . GINS2 regulates the proliferation and apoptosis of colon cancer cells through PTP4A1. Mol Med Rep. 2022;25(4):1–9. 35137928 10.3892/mmr.2022.12633 PMC8855163

[47] McQueeney KE , Salamoun JM , Burnett JC , Barabutis N , Pekic P , Lewandowski SL , et al. Targeting ovarian cancer and endothelium with an allosteric PTP4A3 phosphatase inhibitor. Oncotarget. 2018;9(9):8223–8240. 29492190 10.18632/oncotarget.23787 PMC5823565

[48] Ming J , Liu N , Gu Y , Qiu X , Wang EH . PRL‐3 facilitates angiogenesis and metastasis by increasing ERK phosphorylation and up‐regulating the levels and activities of rho‐A/C in lung cancer. Pathology. 2009;41(2):118–126. 19152186 10.1080/00313020802579268

[49] Gao J , Wang Z , Fu J , Ohno Y , Xu C . Combination treatment with cisplatin, paclitaxel and olaparib has synergistic and dose reduction potential in ovarian cancer cells. Exp Ther Med. 2021;22(3):935. 34335884 10.3892/etm.2021.10367 PMC8290430

[50] Kim SI , Kim JW . Role of surgery and hyperthermic intraperitoneal chemotherapy in ovarian cancer. ESMO Open. 2021;6(3):100149. 33984680 10.1016/j.esmoop.2021.100149 PMC8314869

[51] Grunewald T , Ledermann JA . Targeted therapies for ovarian cancer. Best Pract Res Clin Obstet Gynaecol. 2017;41:139–152. 28111228 10.1016/j.bpobgyn.2016.12.001

[52] Brown Y , Hua S , Tanwar PS . Extracellular matrix in high‐grade serous ovarian cancer: advances in understanding of carcinogenesis and cancer biology. Matrix Biol. 2023;118:16–46. 36781087 10.1016/j.matbio.2023.02.004

[53] Wang Y , Guo Y , Hu Y , Sun Y , Xu D . Endosulfan triggers epithelial‐mesenchymal transition via PTP4A3‐mediated TGF‐beta signaling pathway in prostate cancer cells. Sci Total Environ. 2020;731:139234. 32413665 10.1016/j.scitotenv.2020.139234

[54] Degirmenci U , Wang M , Hu J . Targeting aberrant RAS/RAF/MEK/ERK signaling for cancer therapy. Cells. 2020;9(1):1–33. 10.3390/cells9010198 PMC7017232 31941155

[55] Dunnett‐Kane V , Burkitt‐Wright E , Blackhall FH , Malliri A , Evans DG , Lindsay CR . Germline and sporadic cancers driven by the RAS pathway: parallels and contrasts. Ann Oncol. 2020;31(7):873–883. 32240795 10.1016/j.annonc.2020.03.291 PMC7322396

[56] Dienstmann R , Rodon J , Serra V , Tabernero J . Picking the point of inhibition: a comparative review of PI3K/AKT/mTOR pathway inhibitors. Mol Cancer Ther. 2014;13(5):1021–1031. 24748656 10.1158/1535-7163.MCT-13-0639

[57] Ye Z , al‐aidaroos AQO , Park JE , Yuen HF , Zhang SD , Gupta A , et al. PRL‐3 activates mTORC1 in cancer progression. Sci Rep. 2015;5:17046. 26597054 10.1038/srep17046 PMC4657013

[58] Wang L , Liu J , Zhong Z , Gong X , Liu W , Shi L , et al. PTP4A3 is a target for inhibition of cell proliferatin, migration and invasion through Akt/mTOR signaling pathway in glioblastoma under the regulation of miR‐137. Brain Res. 2016;1646:441–450. 27328425 10.1016/j.brainres.2016.06.026

[59] Csoboz B , Gombos I , Tatrai E , Tovari J , Kiss AL , Horvath I , et al. Chemotherapy induced PRL3 expression promotes cancer growth via plasma membrane remodeling and specific alterations of caveolae‐associated signaling. Cell Commun Signal. 2018;16(1):51. 30157875 10.1186/s12964-018-0264-8 PMC6116440

[60] Lazo JS , Sharlow ER , Cornelison R , Hart DJ , Llaneza DC , Mendelson AJ , et al. Credentialing and pharmacologically targeting PTP4A3 phosphatase as a molecular target for ovarian cancer. Biomolecules. 2021;11(7):1–15. 10.3390/biom11070969 PMC8329922 34209460

