# Supplementary material for: Targeting of PTP4A3 overexpression sensitises HGSOC cells towards chemotherapeutic drugs
Source: Mol Oncol. 2025 Jul 14;19(11):3427–44. doi: 10.1002/1878-0261.70092 (PMC12591308; doi:10.1002/1878-0261.70092)
Supplement: Supplementary file 1 — Fig. S1. IC50 curves for JMS‐053, 5FU, CDDP and PTX. Fig. S2. Kuramochi cells show higher sensitivity to the pan‐PTP4A/PRL inhibitor (iPRL) than OVCAR 3 and OVCAR 4 cells. Fig. S3. Kuramochi‐KD (K‐KD) cells show higher sensitivity to PRL inhibitor (iPRL) than K‐Scr cells, however, K‐Scr shows higher resistance than K‐WT cells. Fig. S4. OVCAR 4‐KD (4‐KD) cells show higher sensitivity to the PRL inhibitor (iPRL) than 4‐WT and 4‐Scr cells. Fig. S5. Kuramochi cells show higher sensitivity to 5FU than OVCAR 3 and OVCAR 4. Fig. S6. Kuramochi‐KD (K‐KD) cells show higher sensitivity to 5FU than K‐Scr, however, K‐Scr shows higher resistance than K‐WT. Fig. S7. OVCAR 4‐KD (4‐KD) cells show higher sensitivity to 5FU than 4‐WT and 4‐Scr. Fig. S8. OVCAR 3 cells show higher sensitivity to cisplatin (CDDP) than OVCAR 4 and Kuramochi. Fig. S9. Kuramochi‐KD (K‐KD) cells show higher sensitivity to cisplatin (CDDP) than K‐WT and K‐Scr. Fig. S10. PTP4A3 silencing does not produce a significant effect in the response of OVCAR 4 cells to cisplatin (CDDP) treatment. Fig. S11. OVCAR 3 cells show higher sensitivity to paclitaxel (PTX) than OVCAR 4 and Kuramochi. Fig. S12. Kuramochi‐KD (K‐KD) cells show higher sensitivity to paclitaxel (PTX) than K‐Scr, however, K‐WT is the most sensitive. Fig. S13. OVCAR 4‐KD (4‐KD) cells show higher sensitivity to paclitaxel (PTX) than 4‐WT and 4‐Scr. Fig. S14. PTP4A3 mRNA expression in OVCAR 4 and Kuramochi cells upon lentiviral‐mediated shRNA knockdown. [file MOL2-19-3427-s001.zip › Garza et al Supplementary 2025 revised.pdf]

SUPPLEMENTARY FIGURES

Supplementary figure 1. IC50 curves for JMS-053, 5FU, CDDP and PTX. Confluency data at 48 h from the previous experiment were analysed to obtain the IC50s for each drug in Kuramochi and OVCAR 4 comparing shPTP4A3 cell lines against their scrambled control counterparts. Data was pooled from 3 (PTX) or 4 (iPRL, 5FU and CDDP) independent experiments, error bars = ±S.E.M.

Supplementary figure 2. Kuramochi cells show higher sensitivity to the pan-PTP4A/PRL inhibitor (iPRL) than OVCAR 3 and OVCAR 4 cells. Kuramochi, OVCAR 4 and OVCAR 3 cells were treated with increasing iPRL (JMS-053) concentrations (0 to 25 µM) in the presence of 2.5 µg/ml Propidium Iodide (PI) and incubated in the Incucyte for 72 hrs. Phase-contrast and red fluorescence (PI) images were taken every 3 hrs. A) Cell confluence plotted over time relative to initial (0 hr) confluence (set to 100) (left), and the ratio of red fluorescence (PI-positive) to total cell confluence plotted over time (right). Comparative analysis of the 3 HGSOC cell lines treated with 0 µM, 5 µM and 12.5 µM iPRL for B) confluency over 72 hrs and C) 24 hrs for PI/confluency (relative to control (0 hr)). Data was pooled from 3 (Kuramochi) or 4 (OVCAR 3 and OVCAR 4) independent experiments, error bars = ±S.E.M. D) Overlayed phase-contrast and fluorescence images (10x magnification) at 0 and 24 hrs treatment with 0 µM (0.2% DMSO), 5 µM or 12.5 µM iPRL.

Supplementary figure 3. Kuramochi-KD (K-KD) cells show higher sensitivity to PRL inhibitor (iPRL) than K-Scr cells, however, K-Scr shows higher resistance than K-WT cells. K-WT, K-Scr and K-KD cells were treated with increasing iPRL (JMS-053) concentrations (0 to 25 μM) in the presence of 2.5 μg/ml Propidium Iodide (PI) and incubated in the Incucyte for 72 hrs. Phase-contrast and red fluorescence (PI) images were taken every 3 hrs. **A)** Cell confluence plotted over time relative to initial (0 hr) confluence (set to 100) (left), and the ratio of red fluorescence (PI-positive) to total cell confluence plotted over time (right). Comparative analysis of the 3 Kuramochi cell lines treated with 0 μM, 5 μM and 12.5 μM iPRL for: **B)** confluency over 72 hrs and **C)** 24 hrs for PI/confluency (relative to control (0 hr)). Data was pooled from 3 (K-WT) or 4 (K-Scr and K-KD) independent experiments, error bars = ±S.E.M. **D)** Overlayed phase-contrast and fluorescence images (10x magnification) at 0 and 24 hrs treatment with 0 μM (0.2% DMSO), 5 μM or 12.5 μM iPRL.

Supplementary figure 4. OVCAR 4-KD (4-KD) cells show higher sensitivity to the PRL inhibitor (iPRL) than 4-WT and 4-Scr cells. 4-WT, 4-Scr and 4-KD cells were treated with increasing iPRL (JMS-053) concentrations (0 to 25 μM) in the presence of 2.5 μg/ml Propidium Iodide (PI) and incubated in the Incucyte for 72 hrs. Phase-contrast and red fluorescence (PI) images were taken every 3 hrs. **A)** Cell confluence plotted over time relative to initial (0 hr) confluence (set to 100) (left), and the ratio of red fluorescence (PI-positive) to total cell confluence plotted over time (right). Comparative analysis of the 3 OVCAR 4 cell lines treated with 0 μM, 5 μM and 12.5 μM iPRL for: **B)** confluency over 72 hrs and **C)** 24 hrs for PI/confluency (relative to control (0 hr)). Data was pooled from 4 independent

experiments, error bars = ±S.E.M. **D)** Overlayed phase-contrast and fluorescence images (10x magnification) at 0 and 24 hrs treatment with 0 µM (0.2% DMSO), 5 µM or 12.5 µM iPRL.

Supplementary figure 5. Kuramochi cells show higher sensitivity to 5FU than OVCAR 3 and OVCAR 4. Kuramochi, OVCAR 4 and OVCAR 3 cells were treated with increasing 5FU concentrations (0 to 1 mM) in the presence of 2.5 µg/ml Propidium Iodide (PI) and incubated in the Incucyte for 72 hrs. Phase-contrast and red fluorescence (PI) images were taken every 3 hrs. **A)** Cell confluence plotted over time relative to initial (0 hr) confluence (set to 100) (left), and the ratio of red fluorescence (PI-positive) to total cell confluence plotted over time (right). Comparative analysis of the 3 HGSOC cell lines treated with 0 µM, 100 µM and 1000 µM 5FU for: **B)** confluency over 72 hrs and **C)** 24 hrs for PI/confluency (relative to control (0 hr)). Data was pooled from 4 independent experiments, error bars = ±S.E.M. **D)** Overlayed phase-contrast and fluorescence images (10x magnification) at 0 and 24 hrs treatment with 0 µM (0.5% DMSO), 100 µM and 1 mM 5FU.

Supplementary figure 6. Kuramochi-KD (K-KD) cells show higher sensitivity to 5FU than K-Scr, however, K-Scr shows higher resistance than K-WT. K-WT, K-Scr and K-KD cells were treated with increasing 5FU concentrations (0 to 1 mM) in the presence of 2.5 µg/ml Propidium Iodide (PI) and incubated in the Incucyte for 72 hrs. Phase-contrast and red fluorescence (PI) images were taken every 3 hrs. **A)** Cell confluence plotted over time relative to initial (0 hr) confluence

(set to 100) (left), and the ratio of red fluorescence (PI-positive) to total cell confluence plotted over time (right). Comparative analysis of the 3 Kuramochi cell lines treated with 0 µM, 100 µM and 1000 µM 5FU for: **B)** confluency over 72 hrs and **C)** 24 hrs for PI/confluency (relative to control (0 hr)). Data was pooled from 4 independent experiments, error bars = ±S.E.M. **D)** Overlayed phase-contrast and fluorescence images (10x magnification) at 0 and 24 hrs treatment with 0 µM (0.5% DMSO), 100 µM and 1 mM 5FU.

Supplementary figure 7. OVCAR 4-KD (4-KD) cells show higher sensitivity to 5FU than 4-WT and 4-Scr. 4-WT, 4-Scr and 4-KD cells were treated with increasing 5FU concentrations (0 to 1 mM) in the presence of 2.5 µg/ml Propidium Iodide (PI) and incubated in the Incucyte for 72 hrs. Phase-contrast and red fluorescence (PI) images were taken every 3 hrs. **A)** Cell confluence plotted over time relative to initial (0 hr) confluence (set to 100) (left), and the ratio of red fluorescence (PI-positive) to total cell confluence plotted over time (right). Comparative analysis of the 3 OVCAR 4 cell lines treated with 0 µM, 100 µM and 1000 µM 5FU for: **B)** confluency over 72 hrs and **C)** 24 hrs for PI/confluency (relative to control (0 hr)). Data was pooled from 4 independent experiments, error bars = ±S.E.M. **D)** Overlayed phase-contrast and fluorescence images (10x magnification) at 0 and 24 hrs treatment with 0 µM (0.5% DMSO), 100 µM and 1 mM 5FU.

Supplementary figure 8. OVCAR 3 cells show higher sensitivity to cisplatin (CDDP) than OVCAR 4 and Kuramochi. Kuramochi, OVCAR 4 and OVCAR 3

cells were treated with increasing CDDP concentrations (0 to 40 µM) in the presence of 2.5 µg/ml Propidium Iodide (PI) and incubated in the Incucyte for 72 hrs. Phase-contrast and red fluorescence (PI) images were taken every 3 hrs. **A)** Cell confluence plotted over time relative to initial (0 hr) confluence (set to 100) (left), and the ratio of red fluorescence (PI-positive) to total cell confluence plotted over time (right). Comparative analysis of the 3 HGSOC cell lines treated with 0 µM, 10 µM and 20 µM CDDP for: **B)** confluency over 72 hrs and **C)** 24 hrs for PI/confluency (relative to control (0 hr)). Data was pooled from 4 independent experiments, error bars = ±S.E.M. **D)** Overlayed phase-contrast and fluorescence images (10x magnification) at 0 and 24 hrs treatment with 0 µM (5% of 0.9% NaCl), 10 µM and 20 µM CDDP.

Supplementary figure 9. Kuramochi-KD (K-KD) cells show higher sensitivity to cisplatin (CDDP) than K-WT and K-Scr. K-WT, K-Scr and K-KD cells were treated with increasing CDDP concentrations (0 to 40 µM) in the presence of 2.5 µg/ml Propidium Iodide (PI) and incubated in the Incucyte for 72 hrs. Phase-contrast and red fluorescence (PI) images were taken every 3 hrs. **A)** Cell confluence plotted over time relative to initial (0 hr) confluence (set to 100) (left), and the ratio of red fluorescence (PI-positive) to total cell confluence plotted over time (right). Comparative analysis of the 3 Kuramochi cell lines treated with 0 µM, 10 µM and 20 µM CDDP for: **B)** confluency over 72 hrs and **C)** 24 hrs for PI/confluency (relative to control (0 hr)). Data was pooled from 4 independent experiments, error bars = ±S.E.M. **D)** Overlayed phase-contrast and fluorescence images (10x magnification) at 0 and 24 hrs treatment with 0 µM (5% of 0.9% NaCl), 10 µM and 20 µM CDDP.

Supplementary figure 10. *PTP4A3* silencing does not produce a significant effect in the response of OVCAR 4 cells to cisplatin (CDDP) treatment. 4-WT, 4-Scr and 4-KD cells were treated with increasing CDDP concentrations (0 to 40 µM) in the presence of 2.5 µg/ml Propidium Iodide (PI) and incubated in the Incucyte for 72 hrs. Phase-contrast and red fluorescence (PI) images were taken every 3 hrs. **A)** Cell confluence plotted over time relative to initial (0 hr) confluence (set to 100) (left), and the ratio of red fluorescence (PI-positive) to total cell confluence plotted over time (right). Comparative analysis of the 3 OVCAR 4 cell lines treated with 0 µM, 10 µM and 20 µM CDDP for: **B)** confluency over 72 hrs and **C)** 24 hrs for PI/confluency (relative to control (0 hr)). Data was pooled from 4 independent experiments, error bars = ±S.E.M. **D)** Overlayed phase-contrast and fluorescence images (10x magnification) at 0 and 24 hrs treatment with 0 µM (5% of 0.9% NaCl), 10 µM and 20 µM CDDP.

Supplementary figure 11. OVCAR 3 cells show higher sensitivity to paclitaxel (PTX) than OVCAR 4 and Kuramochi. Kuramochi, OVCAR 4 and OVCAR 3 cells were treated with increasing PTX concentrations (0 to 100 nM) in the presence of 2.5 µg/ml Propidium Iodide (PI) and incubated in the Incucyte for 72 hrs. Phase-contrast and red fluorescence (PI) images were taken every 3 hrs. **A)** Cell confluence plotted over time relative to initial (0 hr) confluence (set to 100) (left), and the ratio of red fluorescence (PI-positive) to total cell confluence plotted over time (right). Comparative analysis of the 3 HGSOC cell lines treated with 0 nM, 0.5 nM and 10 nM PTX for: **B)** confluency over 72 hrs and **C)** 24 hrs for PI/confluency (relative to control (0 hr)). Data was pooled from 3 independent

experiments, error bars = ±S.E.M. **D)** Overlayed phase-contrast and fluorescence images (10x magnification) at 0 and 24 hrs treatment with 0 nM (0.1% DMSO), 0.5 nM and 10 nM PTX.

Supplementary figure 12. Kuramochi-KD (K-KD) cells show higher sensitivity to paclitaxel (PTX) than K-Scr, however, K-WT is the most sensitive. K-WT, K-Scr and K-KD cells were treated with increasing PTX concentrations (0 to 100 nM) in the presence of 2.5 µg/ml Propidium Iodide (PI) and incubated in the Incucyte for 72 hrs. Phase-contrast and red fluorescence (PI) images were taken every 3 hrs. **A)** Cell confluence plotted over time relative to initial (0 hr) confluence (set to 100) (left), and the ratio of red fluorescence (PI-positive) to total cell confluence plotted over time (right). Comparative analysis of the 3 Kuramochi cell lines treated with 0 nM, 0.5 nM and 10 nM PTX for: **B)** confluency over 72 hrs and **C)** 24 hrs for PI/confluency (relative to control (0 hr)). Data was pooled from 3 independent experiments, error bars = ±S.E.M. **D)** Overlayed phase-contrast and fluorescence images (10x magnification) at 0 and 24 hrs treatment with 0 nM (0.1% DMSO), 0.5 nM and 10 nM PTX.

Supplementary figure 13. OVCAR 4-KD (4-KD) cells show higher sensitivity to paclitaxel (PTX) than 4-WT and 4-Scr. 4-WT, 4-Scr and 4-KD cells were treated with increasing PTX concentrations (0 to 100 nM) in the presence of 2.5 µg/ml Propidium Iodide (PI) and incubated in the Incucyte for 72 hrs. Phase-contrast and red fluorescence (PI) images were taken every 3 hrs. **A)** Cell confluence plotted over time relative to initial (0 hr) confluence (set to 100) (left),

and the ratio of red fluorescence (PI-positive) to total cell confluence plotted over time (right). Comparative analysis of the 3 OVCAR 4 cell lines treated with 0 nM, 0.5 nM and 10 nM PTX for: **B)** confluency over 72 hrs and **C)** 24 hrs for PI/confluency (relative to control (0 hr)). Data was pooled from 3 independent experiments, error bars = ±S.E.M. **D)** Overlayed phase-contrast and fluorescence images (10x magnification) at 0 and 24 hrs treatment with 0 nM (0.1% DMSO), 0.5 nM and 10 nM PTX.

Supplementary figure 14. PTP4A3 mRNA expression in OVCAR 4 and Kuramochi cells upon lentiviral mediated shRNA knockdown. RNA from OVCAR 4 (top) and Kuramochi (bottom) WT, Scrambled and Knockdowns (1883 and 1884) cell lysates were isolated and analysed by qPCR. PTP4A3 expression levels were normalised to GAPDH and are depicted as a fold increase. Data represents three independent experiments, error bars = ± S.E.M.
