# Supplementary figures and images for: Targeting of PTP4A3 overexpression sensitises HGSOC cells towards chemotherapeutic drugs
Source: Mol Oncol. 2025 Jul 14;19(11):3427–44. doi: 10.1002/1878-0261.70092 (PMC12591308; doi:10.1002/1878-0261.70092)

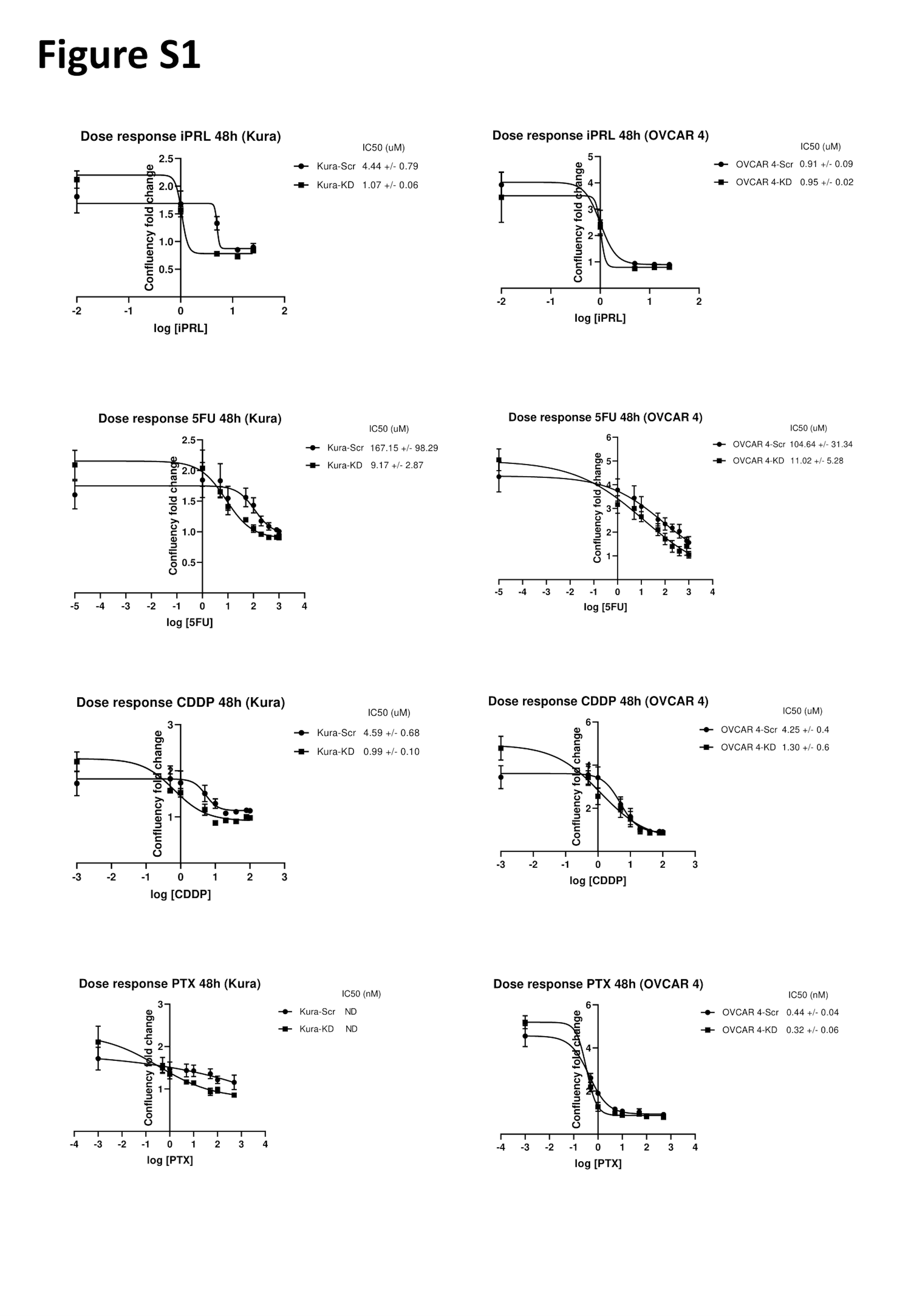

Supplement: Supplementary file 1 — Fig. S1. IC50 curves for JMS‐053, 5FU, CDDP and PTX. Fig. S2. Kuramochi cells show higher sensitivity to the pan‐PTP4A/PRL inhibitor (iPRL) than OVCAR 3 and OVCAR 4 cells. Fig. S3. Kuramochi‐KD (K‐KD) cells show higher sensitivity to PRL inhibitor (iPRL) than K‐Scr cells, however, K‐Scr shows higher resistance than K‐WT cells. Fig. S4. OVCAR 4‐KD (4‐KD) cells show higher sensitivity to the PRL inhibitor (iPRL) than 4‐WT and 4‐Scr cells. Fig. S5. Kuramochi cells show higher sensitivity to 5FU than OVCAR 3 and OVCAR 4. Fig. S6. Kuramochi‐KD (K‐KD) cells show higher sensitivity to 5FU than K‐Scr, however, K‐Scr shows higher resistance than K‐WT. Fig. S7. OVCAR 4‐KD (4‐KD) cells show higher sensitivity to 5FU than 4‐WT and 4‐Scr. Fig. S8. OVCAR 3 cells show higher sensitivity to cisplatin (CDDP) than OVCAR 4 and Kuramochi. Fig. S9. Kuramochi‐KD (K‐KD) cells show higher sensitivity to cisplatin (CDDP) than K‐WT and K‐Scr. Fig. S10. PTP4A3 silencing does not produce a significant effect in the response of OVCAR 4 cells to cisplatin (CDDP) treatment. Fig. S11. OVCAR 3 cells show higher sensitivity to paclitaxel (PTX) than OVCAR 4 and Kuramochi. Fig. S12. Kuramochi‐KD (K‐KD) cells show higher sensitivity to paclitaxel (PTX) than K‐Scr, however, K‐WT is the most sensitive. Fig. S13. OVCAR 4‐KD (4‐KD) cells show higher sensitivity to paclitaxel (PTX) than 4‐WT and 4‐Scr. Fig. S14. PTP4A3 mRNA expression in OVCAR 4 and Kuramochi cells upon lentiviral‐mediated shRNA knockdown. [file MOL2-19-3427-s001.zip › Figure S1 - revised.tiff]

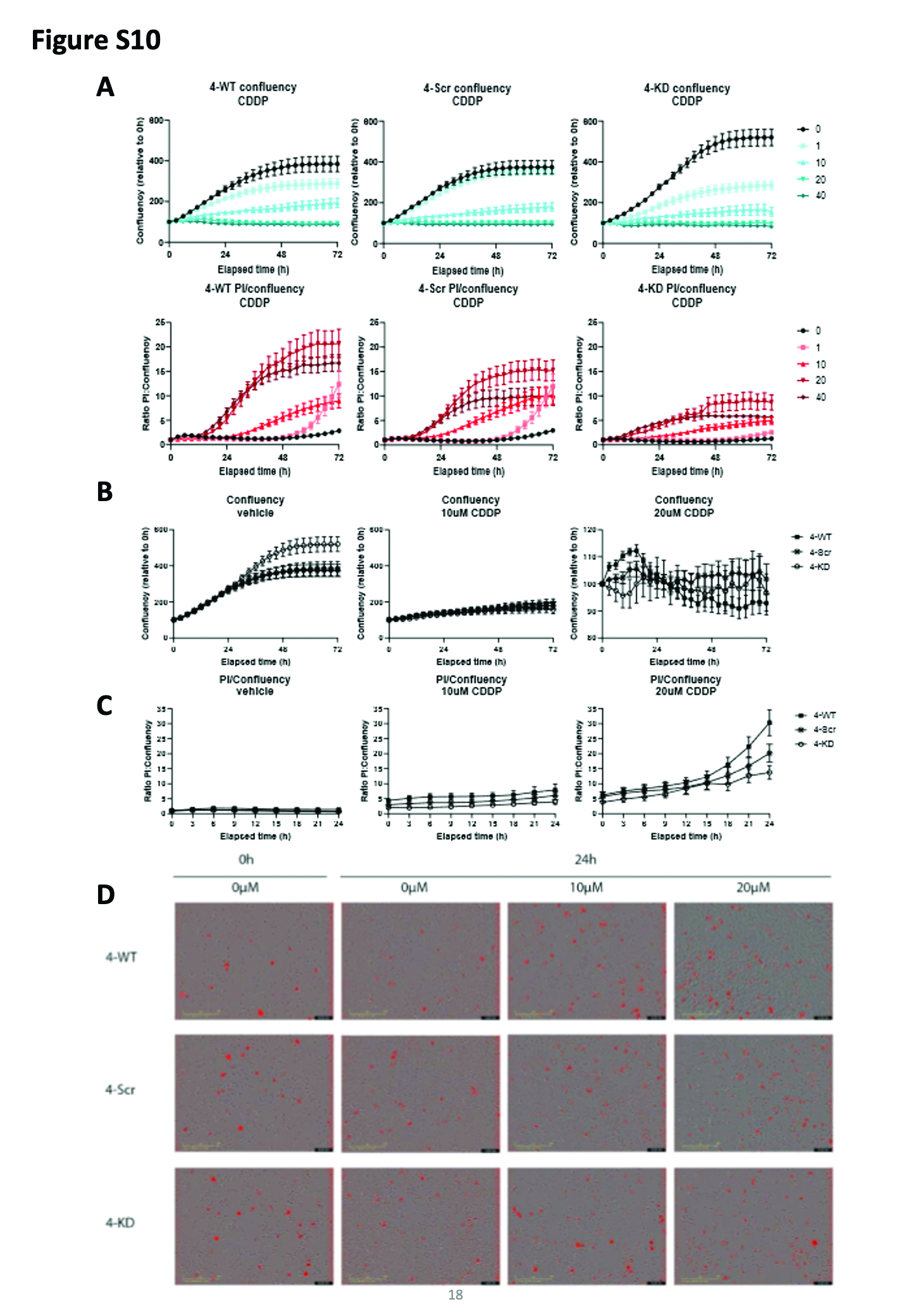

Supplement: Supplementary file 1 — Fig. S1. IC50 curves for JMS‐053, 5FU, CDDP and PTX. Fig. S2. Kuramochi cells show higher sensitivity to the pan‐PTP4A/PRL inhibitor (iPRL) than OVCAR 3 and OVCAR 4 cells. Fig. S3. Kuramochi‐KD (K‐KD) cells show higher sensitivity to PRL inhibitor (iPRL) than K‐Scr cells, however, K‐Scr shows higher resistance than K‐WT cells. Fig. S4. OVCAR 4‐KD (4‐KD) cells show higher sensitivity to the PRL inhibitor (iPRL) than 4‐WT and 4‐Scr cells. Fig. S5. Kuramochi cells show higher sensitivity to 5FU than OVCAR 3 and OVCAR 4. Fig. S6. Kuramochi‐KD (K‐KD) cells show higher sensitivity to 5FU than K‐Scr, however, K‐Scr shows higher resistance than K‐WT. Fig. S7. OVCAR 4‐KD (4‐KD) cells show higher sensitivity to 5FU than 4‐WT and 4‐Scr. Fig. S8. OVCAR 3 cells show higher sensitivity to cisplatin (CDDP) than OVCAR 4 and Kuramochi. Fig. S9. Kuramochi‐KD (K‐KD) cells show higher sensitivity to cisplatin (CDDP) than K‐WT and K‐Scr. Fig. S10. PTP4A3 silencing does not produce a significant effect in the response of OVCAR 4 cells to cisplatin (CDDP) treatment. Fig. S11. OVCAR 3 cells show higher sensitivity to paclitaxel (PTX) than OVCAR 4 and Kuramochi. Fig. S12. Kuramochi‐KD (K‐KD) cells show higher sensitivity to paclitaxel (PTX) than K‐Scr, however, K‐WT is the most sensitive. Fig. S13. OVCAR 4‐KD (4‐KD) cells show higher sensitivity to paclitaxel (PTX) than 4‐WT and 4‐Scr. Fig. S14. PTP4A3 mRNA expression in OVCAR 4 and Kuramochi cells upon lentiviral‐mediated shRNA knockdown. [file MOL2-19-3427-s001.zip › Figure S10.tif]

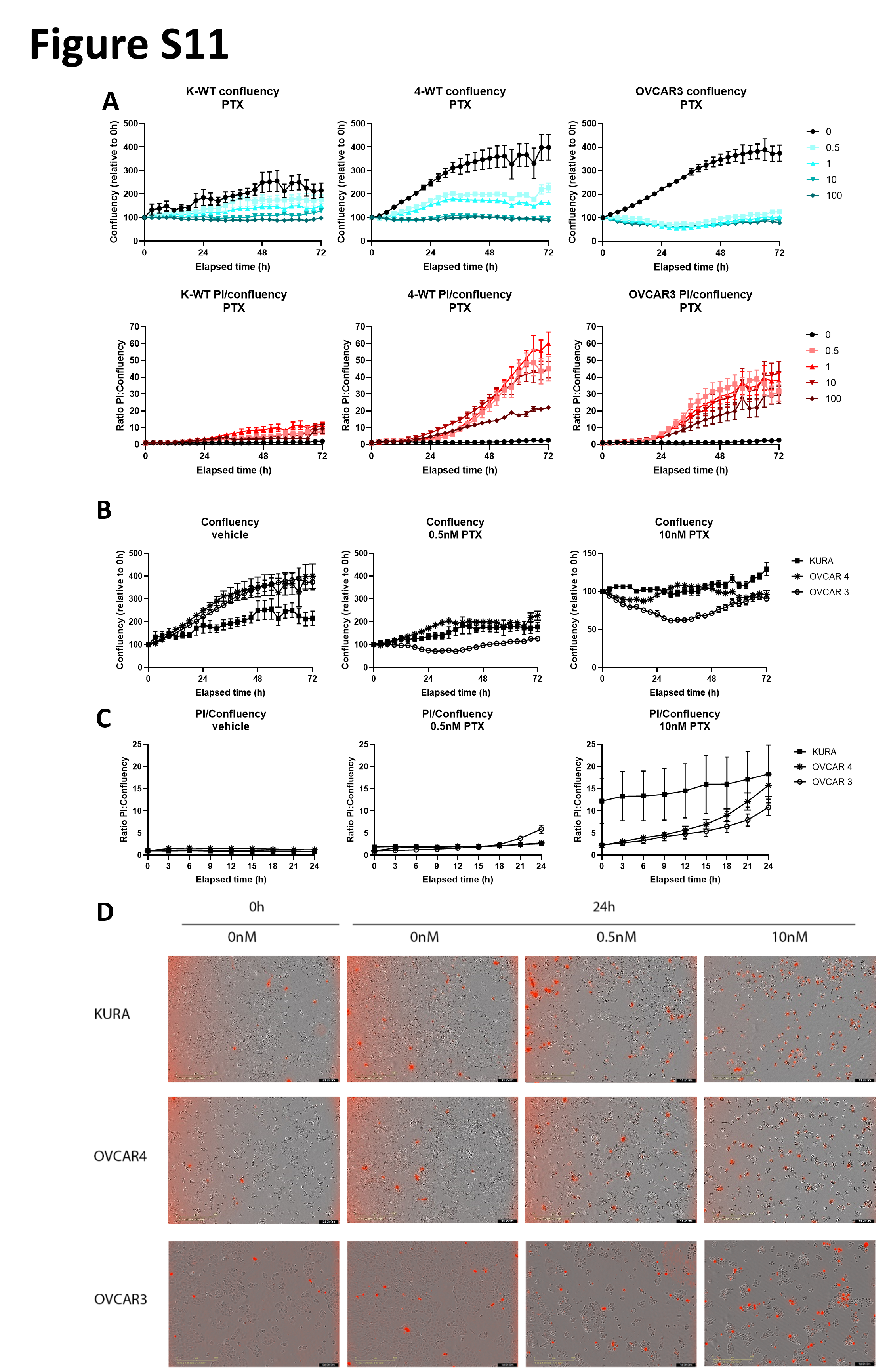

Supplement: Supplementary file 1 — Fig. S1. IC50 curves for JMS‐053, 5FU, CDDP and PTX. Fig. S2. Kuramochi cells show higher sensitivity to the pan‐PTP4A/PRL inhibitor (iPRL) than OVCAR 3 and OVCAR 4 cells. Fig. S3. Kuramochi‐KD (K‐KD) cells show higher sensitivity to PRL inhibitor (iPRL) than K‐Scr cells, however, K‐Scr shows higher resistance than K‐WT cells. Fig. S4. OVCAR 4‐KD (4‐KD) cells show higher sensitivity to the PRL inhibitor (iPRL) than 4‐WT and 4‐Scr cells. Fig. S5. Kuramochi cells show higher sensitivity to 5FU than OVCAR 3 and OVCAR 4. Fig. S6. Kuramochi‐KD (K‐KD) cells show higher sensitivity to 5FU than K‐Scr, however, K‐Scr shows higher resistance than K‐WT. Fig. S7. OVCAR 4‐KD (4‐KD) cells show higher sensitivity to 5FU than 4‐WT and 4‐Scr. Fig. S8. OVCAR 3 cells show higher sensitivity to cisplatin (CDDP) than OVCAR 4 and Kuramochi. Fig. S9. Kuramochi‐KD (K‐KD) cells show higher sensitivity to cisplatin (CDDP) than K‐WT and K‐Scr. Fig. S10. PTP4A3 silencing does not produce a significant effect in the response of OVCAR 4 cells to cisplatin (CDDP) treatment. Fig. S11. OVCAR 3 cells show higher sensitivity to paclitaxel (PTX) than OVCAR 4 and Kuramochi. Fig. S12. Kuramochi‐KD (K‐KD) cells show higher sensitivity to paclitaxel (PTX) than K‐Scr, however, K‐WT is the most sensitive. Fig. S13. OVCAR 4‐KD (4‐KD) cells show higher sensitivity to paclitaxel (PTX) than 4‐WT and 4‐Scr. Fig. S14. PTP4A3 mRNA expression in OVCAR 4 and Kuramochi cells upon lentiviral‐mediated shRNA knockdown. [file MOL2-19-3427-s001.zip › Figure S11 - revised.tif]

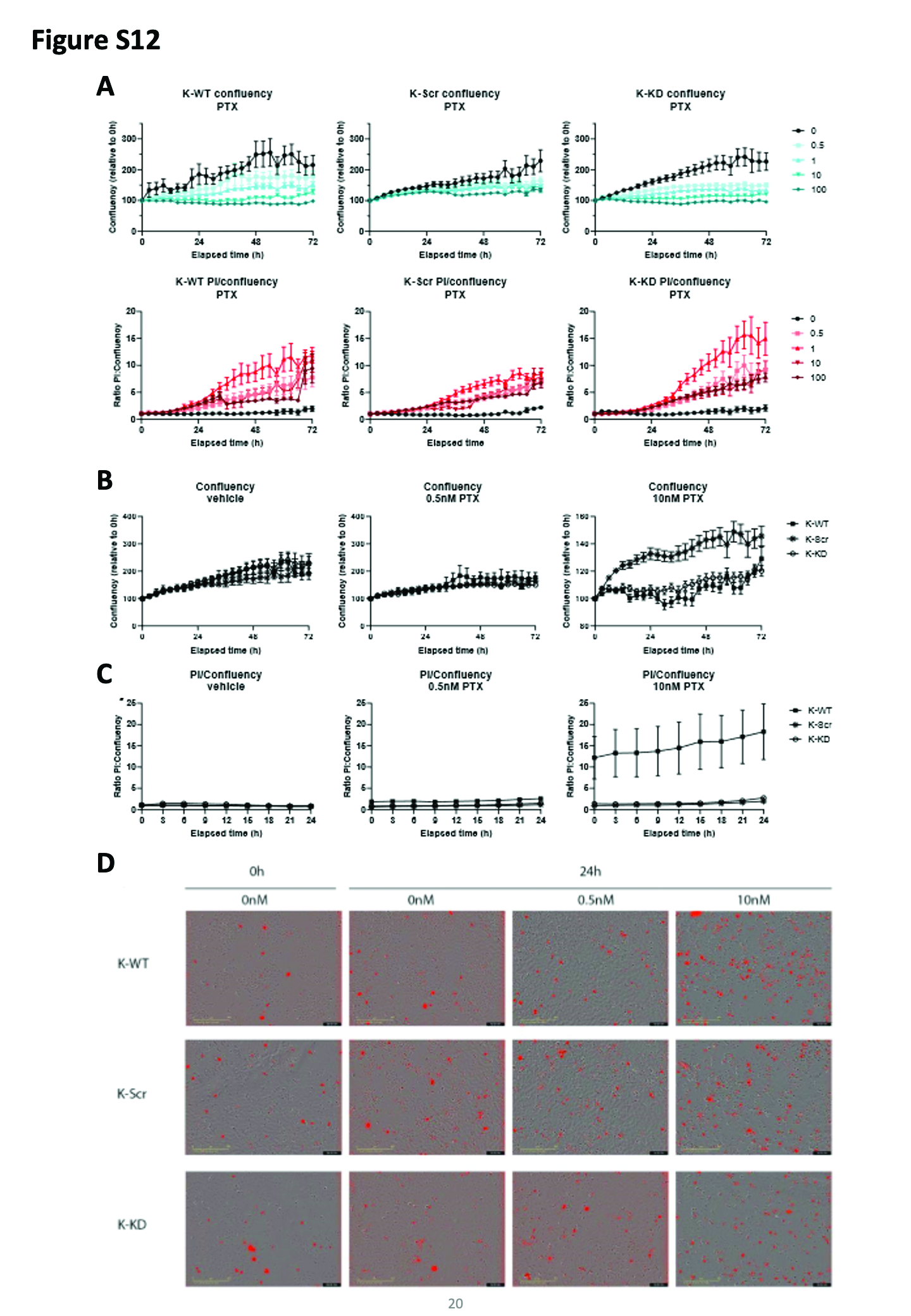

Supplement: Supplementary file 1 — Fig. S1. IC50 curves for JMS‐053, 5FU, CDDP and PTX. Fig. S2. Kuramochi cells show higher sensitivity to the pan‐PTP4A/PRL inhibitor (iPRL) than OVCAR 3 and OVCAR 4 cells. Fig. S3. Kuramochi‐KD (K‐KD) cells show higher sensitivity to PRL inhibitor (iPRL) than K‐Scr cells, however, K‐Scr shows higher resistance than K‐WT cells. Fig. S4. OVCAR 4‐KD (4‐KD) cells show higher sensitivity to the PRL inhibitor (iPRL) than 4‐WT and 4‐Scr cells. Fig. S5. Kuramochi cells show higher sensitivity to 5FU than OVCAR 3 and OVCAR 4. Fig. S6. Kuramochi‐KD (K‐KD) cells show higher sensitivity to 5FU than K‐Scr, however, K‐Scr shows higher resistance than K‐WT. Fig. S7. OVCAR 4‐KD (4‐KD) cells show higher sensitivity to 5FU than 4‐WT and 4‐Scr. Fig. S8. OVCAR 3 cells show higher sensitivity to cisplatin (CDDP) than OVCAR 4 and Kuramochi. Fig. S9. Kuramochi‐KD (K‐KD) cells show higher sensitivity to cisplatin (CDDP) than K‐WT and K‐Scr. Fig. S10. PTP4A3 silencing does not produce a significant effect in the response of OVCAR 4 cells to cisplatin (CDDP) treatment. Fig. S11. OVCAR 3 cells show higher sensitivity to paclitaxel (PTX) than OVCAR 4 and Kuramochi. Fig. S12. Kuramochi‐KD (K‐KD) cells show higher sensitivity to paclitaxel (PTX) than K‐Scr, however, K‐WT is the most sensitive. Fig. S13. OVCAR 4‐KD (4‐KD) cells show higher sensitivity to paclitaxel (PTX) than 4‐WT and 4‐Scr. Fig. S14. PTP4A3 mRNA expression in OVCAR 4 and Kuramochi cells upon lentiviral‐mediated shRNA knockdown. [file MOL2-19-3427-s001.zip › Figure S12.tif]

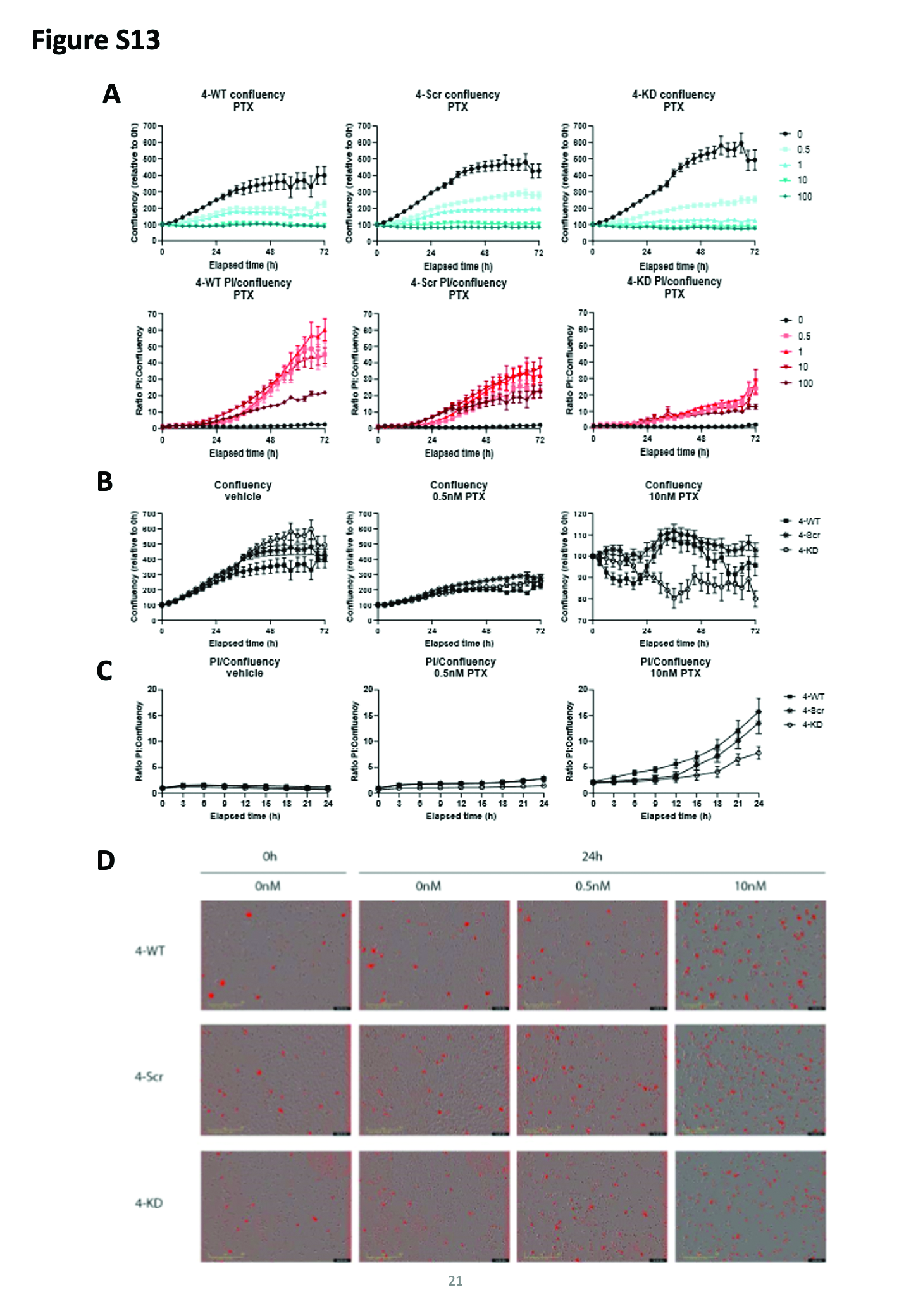

Supplement: Supplementary file 1 — Fig. S1. IC50 curves for JMS‐053, 5FU, CDDP and PTX. Fig. S2. Kuramochi cells show higher sensitivity to the pan‐PTP4A/PRL inhibitor (iPRL) than OVCAR 3 and OVCAR 4 cells. Fig. S3. Kuramochi‐KD (K‐KD) cells show higher sensitivity to PRL inhibitor (iPRL) than K‐Scr cells, however, K‐Scr shows higher resistance than K‐WT cells. Fig. S4. OVCAR 4‐KD (4‐KD) cells show higher sensitivity to the PRL inhibitor (iPRL) than 4‐WT and 4‐Scr cells. Fig. S5. Kuramochi cells show higher sensitivity to 5FU than OVCAR 3 and OVCAR 4. Fig. S6. Kuramochi‐KD (K‐KD) cells show higher sensitivity to 5FU than K‐Scr, however, K‐Scr shows higher resistance than K‐WT. Fig. S7. OVCAR 4‐KD (4‐KD) cells show higher sensitivity to 5FU than 4‐WT and 4‐Scr. Fig. S8. OVCAR 3 cells show higher sensitivity to cisplatin (CDDP) than OVCAR 4 and Kuramochi. Fig. S9. Kuramochi‐KD (K‐KD) cells show higher sensitivity to cisplatin (CDDP) than K‐WT and K‐Scr. Fig. S10. PTP4A3 silencing does not produce a significant effect in the response of OVCAR 4 cells to cisplatin (CDDP) treatment. Fig. S11. OVCAR 3 cells show higher sensitivity to paclitaxel (PTX) than OVCAR 4 and Kuramochi. Fig. S12. Kuramochi‐KD (K‐KD) cells show higher sensitivity to paclitaxel (PTX) than K‐Scr, however, K‐WT is the most sensitive. Fig. S13. OVCAR 4‐KD (4‐KD) cells show higher sensitivity to paclitaxel (PTX) than 4‐WT and 4‐Scr. Fig. S14. PTP4A3 mRNA expression in OVCAR 4 and Kuramochi cells upon lentiviral‐mediated shRNA knockdown. [file MOL2-19-3427-s001.zip › Figure S13.tif]

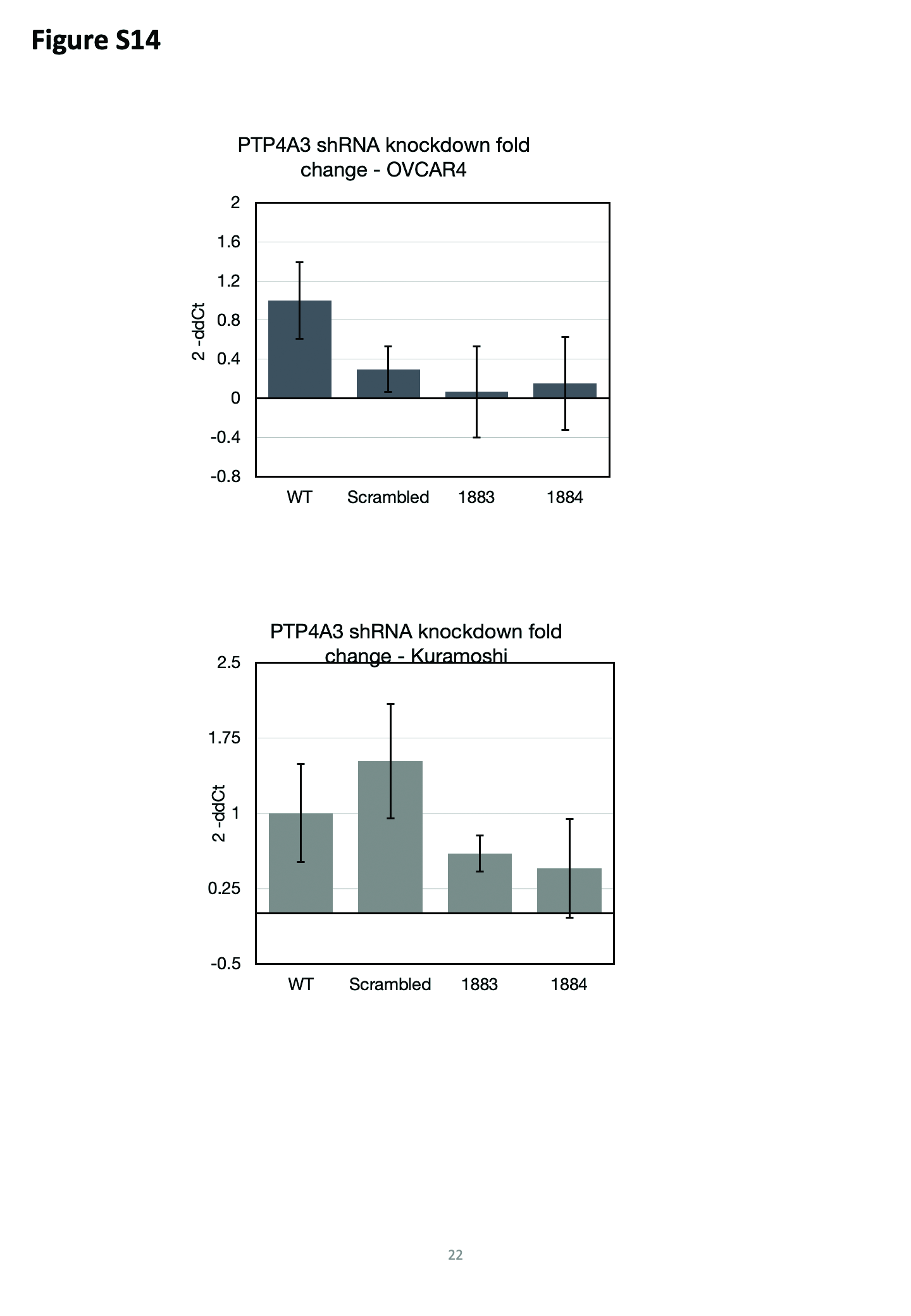

Supplement: Supplementary file 1 — Fig. S1. IC50 curves for JMS‐053, 5FU, CDDP and PTX. Fig. S2. Kuramochi cells show higher sensitivity to the pan‐PTP4A/PRL inhibitor (iPRL) than OVCAR 3 and OVCAR 4 cells. Fig. S3. Kuramochi‐KD (K‐KD) cells show higher sensitivity to PRL inhibitor (iPRL) than K‐Scr cells, however, K‐Scr shows higher resistance than K‐WT cells. Fig. S4. OVCAR 4‐KD (4‐KD) cells show higher sensitivity to the PRL inhibitor (iPRL) than 4‐WT and 4‐Scr cells. Fig. S5. Kuramochi cells show higher sensitivity to 5FU than OVCAR 3 and OVCAR 4. Fig. S6. Kuramochi‐KD (K‐KD) cells show higher sensitivity to 5FU than K‐Scr, however, K‐Scr shows higher resistance than K‐WT. Fig. S7. OVCAR 4‐KD (4‐KD) cells show higher sensitivity to 5FU than 4‐WT and 4‐Scr. Fig. S8. OVCAR 3 cells show higher sensitivity to cisplatin (CDDP) than OVCAR 4 and Kuramochi. Fig. S9. Kuramochi‐KD (K‐KD) cells show higher sensitivity to cisplatin (CDDP) than K‐WT and K‐Scr. Fig. S10. PTP4A3 silencing does not produce a significant effect in the response of OVCAR 4 cells to cisplatin (CDDP) treatment. Fig. S11. OVCAR 3 cells show higher sensitivity to paclitaxel (PTX) than OVCAR 4 and Kuramochi. Fig. S12. Kuramochi‐KD (K‐KD) cells show higher sensitivity to paclitaxel (PTX) than K‐Scr, however, K‐WT is the most sensitive. Fig. S13. OVCAR 4‐KD (4‐KD) cells show higher sensitivity to paclitaxel (PTX) than 4‐WT and 4‐Scr. Fig. S14. PTP4A3 mRNA expression in OVCAR 4 and Kuramochi cells upon lentiviral‐mediated shRNA knockdown. [file MOL2-19-3427-s001.zip › Figure S14.tif]

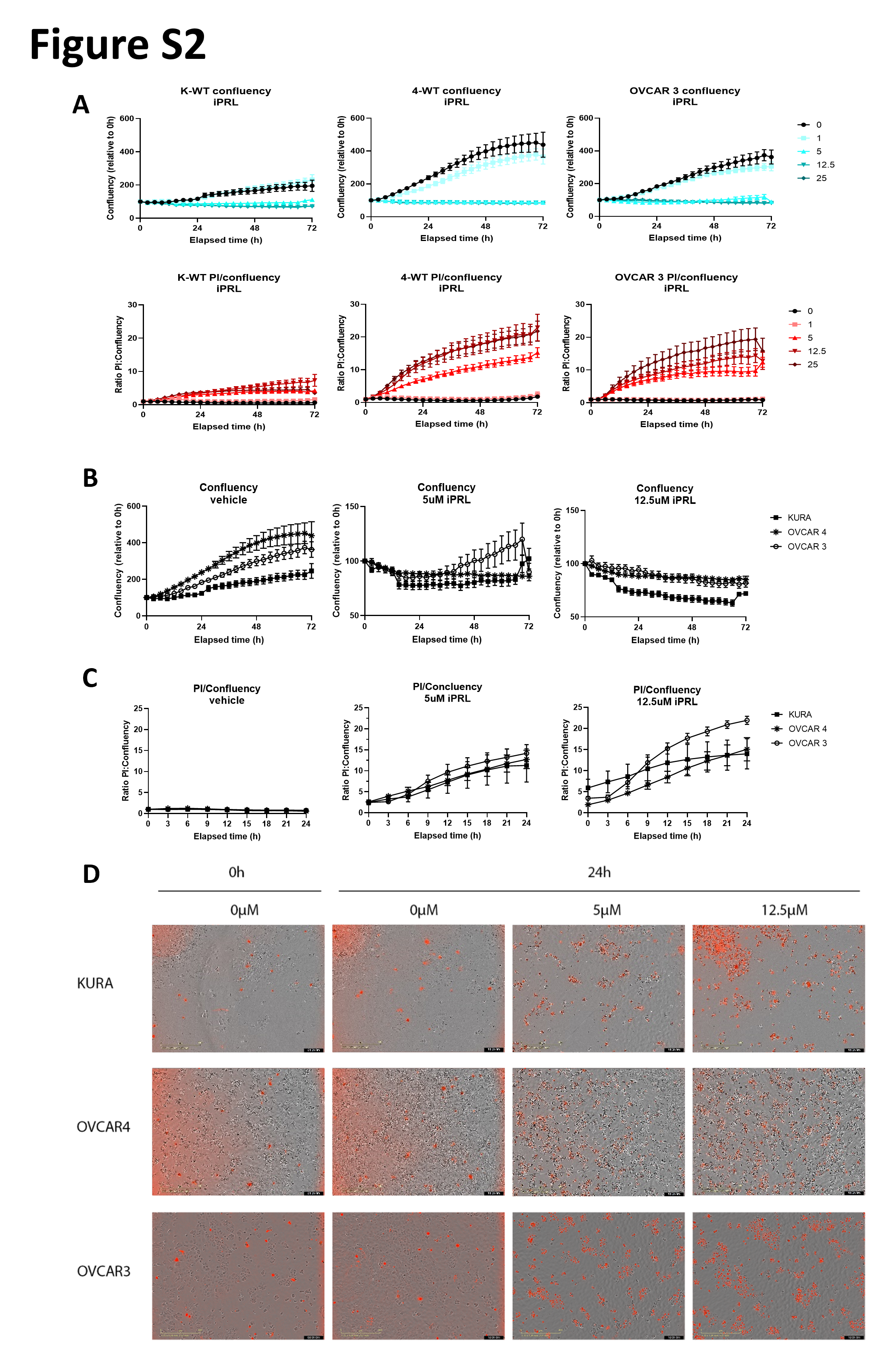

Supplement: Supplementary file 1 — Fig. S1. IC50 curves for JMS‐053, 5FU, CDDP and PTX. Fig. S2. Kuramochi cells show higher sensitivity to the pan‐PTP4A/PRL inhibitor (iPRL) than OVCAR 3 and OVCAR 4 cells. Fig. S3. Kuramochi‐KD (K‐KD) cells show higher sensitivity to PRL inhibitor (iPRL) than K‐Scr cells, however, K‐Scr shows higher resistance than K‐WT cells. Fig. S4. OVCAR 4‐KD (4‐KD) cells show higher sensitivity to the PRL inhibitor (iPRL) than 4‐WT and 4‐Scr cells. Fig. S5. Kuramochi cells show higher sensitivity to 5FU than OVCAR 3 and OVCAR 4. Fig. S6. Kuramochi‐KD (K‐KD) cells show higher sensitivity to 5FU than K‐Scr, however, K‐Scr shows higher resistance than K‐WT. Fig. S7. OVCAR 4‐KD (4‐KD) cells show higher sensitivity to 5FU than 4‐WT and 4‐Scr. Fig. S8. OVCAR 3 cells show higher sensitivity to cisplatin (CDDP) than OVCAR 4 and Kuramochi. Fig. S9. Kuramochi‐KD (K‐KD) cells show higher sensitivity to cisplatin (CDDP) than K‐WT and K‐Scr. Fig. S10. PTP4A3 silencing does not produce a significant effect in the response of OVCAR 4 cells to cisplatin (CDDP) treatment. Fig. S11. OVCAR 3 cells show higher sensitivity to paclitaxel (PTX) than OVCAR 4 and Kuramochi. Fig. S12. Kuramochi‐KD (K‐KD) cells show higher sensitivity to paclitaxel (PTX) than K‐Scr, however, K‐WT is the most sensitive. Fig. S13. OVCAR 4‐KD (4‐KD) cells show higher sensitivity to paclitaxel (PTX) than 4‐WT and 4‐Scr. Fig. S14. PTP4A3 mRNA expression in OVCAR 4 and Kuramochi cells upon lentiviral‐mediated shRNA knockdown. [file MOL2-19-3427-s001.zip › Figure S2 - revised.tif]

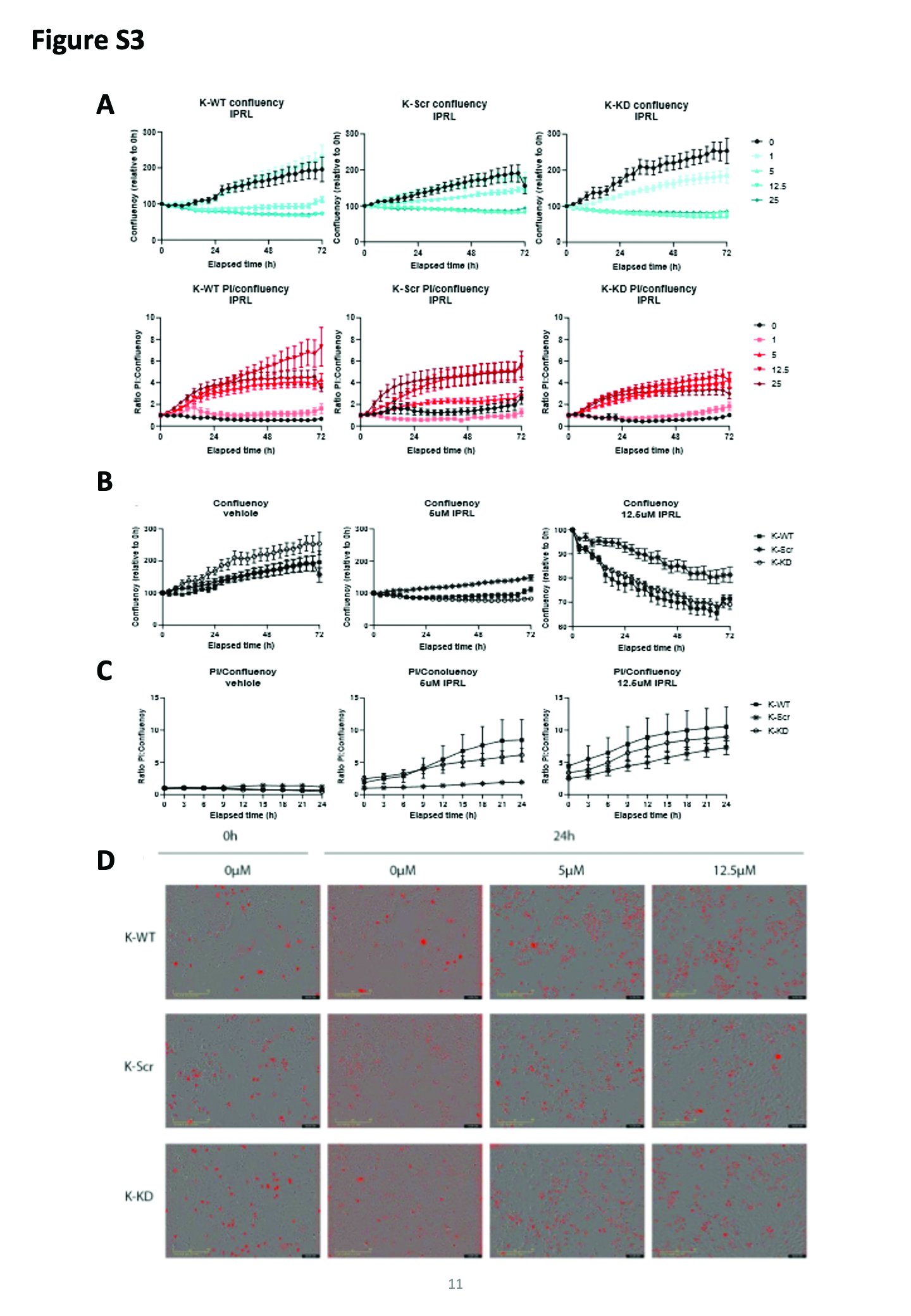

Supplement: Supplementary file 1 — Fig. S1. IC50 curves for JMS‐053, 5FU, CDDP and PTX. Fig. S2. Kuramochi cells show higher sensitivity to the pan‐PTP4A/PRL inhibitor (iPRL) than OVCAR 3 and OVCAR 4 cells. Fig. S3. Kuramochi‐KD (K‐KD) cells show higher sensitivity to PRL inhibitor (iPRL) than K‐Scr cells, however, K‐Scr shows higher resistance than K‐WT cells. Fig. S4. OVCAR 4‐KD (4‐KD) cells show higher sensitivity to the PRL inhibitor (iPRL) than 4‐WT and 4‐Scr cells. Fig. S5. Kuramochi cells show higher sensitivity to 5FU than OVCAR 3 and OVCAR 4. Fig. S6. Kuramochi‐KD (K‐KD) cells show higher sensitivity to 5FU than K‐Scr, however, K‐Scr shows higher resistance than K‐WT. Fig. S7. OVCAR 4‐KD (4‐KD) cells show higher sensitivity to 5FU than 4‐WT and 4‐Scr. Fig. S8. OVCAR 3 cells show higher sensitivity to cisplatin (CDDP) than OVCAR 4 and Kuramochi. Fig. S9. Kuramochi‐KD (K‐KD) cells show higher sensitivity to cisplatin (CDDP) than K‐WT and K‐Scr. Fig. S10. PTP4A3 silencing does not produce a significant effect in the response of OVCAR 4 cells to cisplatin (CDDP) treatment. Fig. S11. OVCAR 3 cells show higher sensitivity to paclitaxel (PTX) than OVCAR 4 and Kuramochi. Fig. S12. Kuramochi‐KD (K‐KD) cells show higher sensitivity to paclitaxel (PTX) than K‐Scr, however, K‐WT is the most sensitive. Fig. S13. OVCAR 4‐KD (4‐KD) cells show higher sensitivity to paclitaxel (PTX) than 4‐WT and 4‐Scr. Fig. S14. PTP4A3 mRNA expression in OVCAR 4 and Kuramochi cells upon lentiviral‐mediated shRNA knockdown. [file MOL2-19-3427-s001.zip › Figure S3.tif]

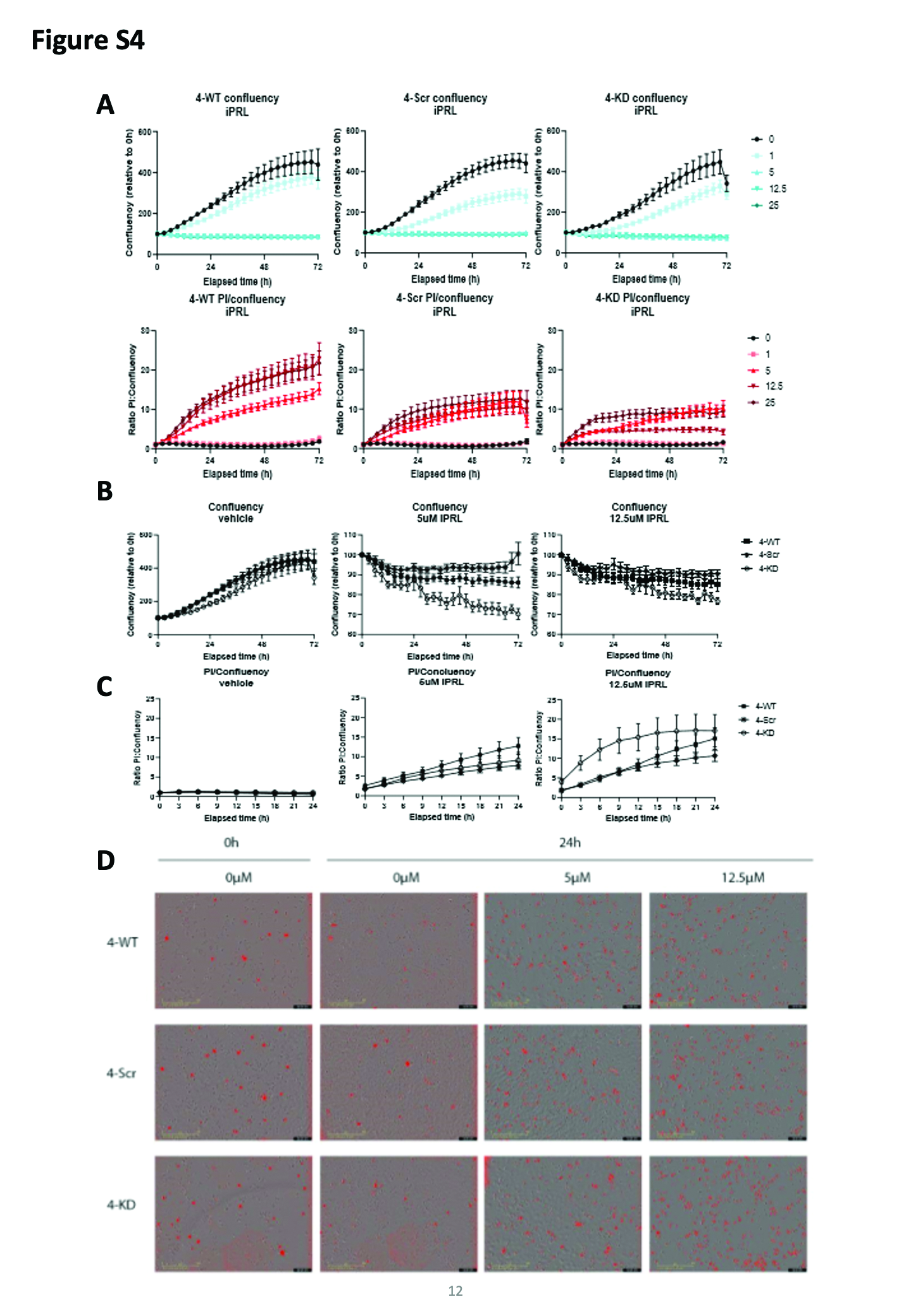

Supplement: Supplementary file 1 — Fig. S1. IC50 curves for JMS‐053, 5FU, CDDP and PTX. Fig. S2. Kuramochi cells show higher sensitivity to the pan‐PTP4A/PRL inhibitor (iPRL) than OVCAR 3 and OVCAR 4 cells. Fig. S3. Kuramochi‐KD (K‐KD) cells show higher sensitivity to PRL inhibitor (iPRL) than K‐Scr cells, however, K‐Scr shows higher resistance than K‐WT cells. Fig. S4. OVCAR 4‐KD (4‐KD) cells show higher sensitivity to the PRL inhibitor (iPRL) than 4‐WT and 4‐Scr cells. Fig. S5. Kuramochi cells show higher sensitivity to 5FU than OVCAR 3 and OVCAR 4. Fig. S6. Kuramochi‐KD (K‐KD) cells show higher sensitivity to 5FU than K‐Scr, however, K‐Scr shows higher resistance than K‐WT. Fig. S7. OVCAR 4‐KD (4‐KD) cells show higher sensitivity to 5FU than 4‐WT and 4‐Scr. Fig. S8. OVCAR 3 cells show higher sensitivity to cisplatin (CDDP) than OVCAR 4 and Kuramochi. Fig. S9. Kuramochi‐KD (K‐KD) cells show higher sensitivity to cisplatin (CDDP) than K‐WT and K‐Scr. Fig. S10. PTP4A3 silencing does not produce a significant effect in the response of OVCAR 4 cells to cisplatin (CDDP) treatment. Fig. S11. OVCAR 3 cells show higher sensitivity to paclitaxel (PTX) than OVCAR 4 and Kuramochi. Fig. S12. Kuramochi‐KD (K‐KD) cells show higher sensitivity to paclitaxel (PTX) than K‐Scr, however, K‐WT is the most sensitive. Fig. S13. OVCAR 4‐KD (4‐KD) cells show higher sensitivity to paclitaxel (PTX) than 4‐WT and 4‐Scr. Fig. S14. PTP4A3 mRNA expression in OVCAR 4 and Kuramochi cells upon lentiviral‐mediated shRNA knockdown. [file MOL2-19-3427-s001.zip › Figure S4.tif]

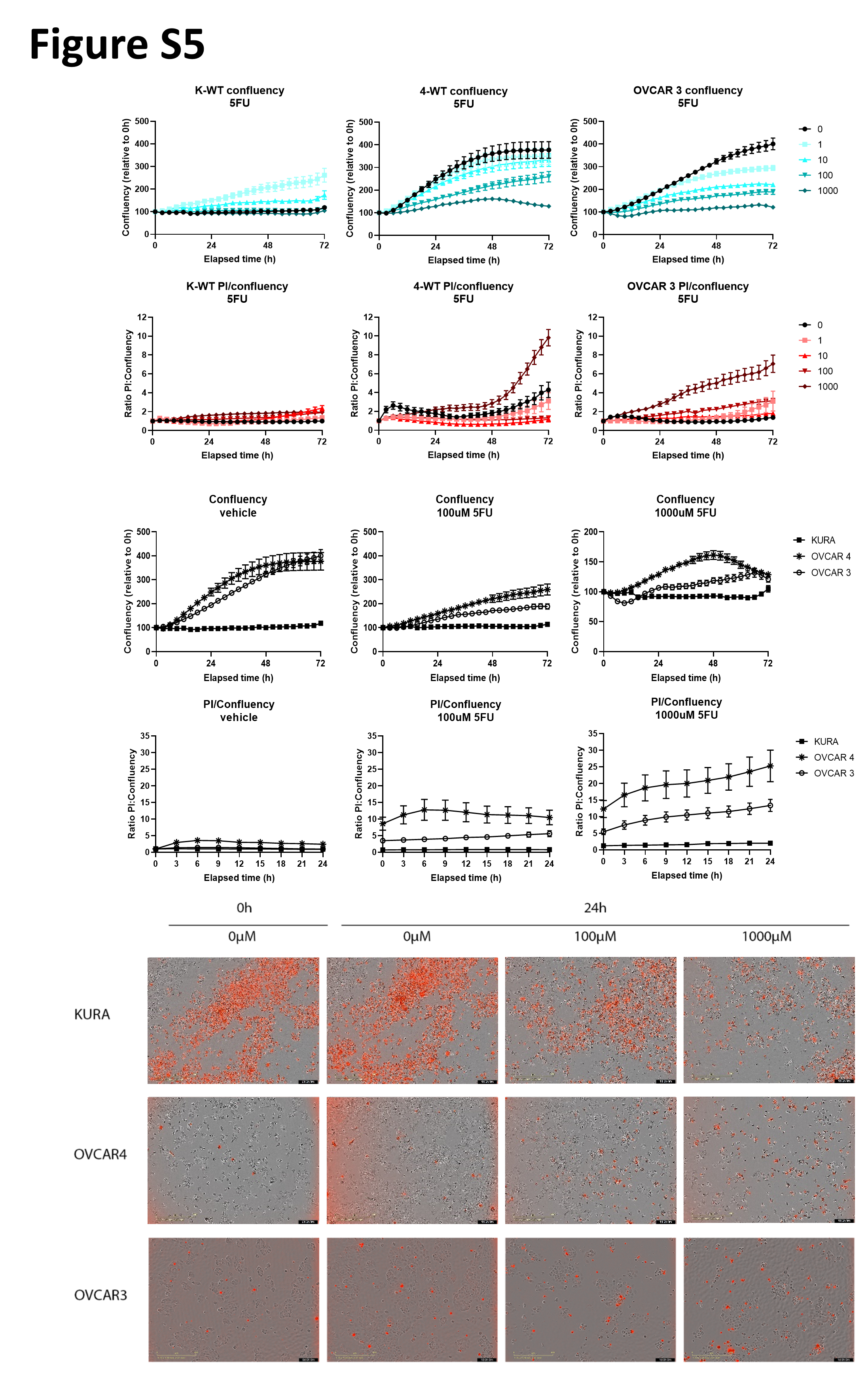

Supplement: Supplementary file 1 — Fig. S1. IC50 curves for JMS‐053, 5FU, CDDP and PTX. Fig. S2. Kuramochi cells show higher sensitivity to the pan‐PTP4A/PRL inhibitor (iPRL) than OVCAR 3 and OVCAR 4 cells. Fig. S3. Kuramochi‐KD (K‐KD) cells show higher sensitivity to PRL inhibitor (iPRL) than K‐Scr cells, however, K‐Scr shows higher resistance than K‐WT cells. Fig. S4. OVCAR 4‐KD (4‐KD) cells show higher sensitivity to the PRL inhibitor (iPRL) than 4‐WT and 4‐Scr cells. Fig. S5. Kuramochi cells show higher sensitivity to 5FU than OVCAR 3 and OVCAR 4. Fig. S6. Kuramochi‐KD (K‐KD) cells show higher sensitivity to 5FU than K‐Scr, however, K‐Scr shows higher resistance than K‐WT. Fig. S7. OVCAR 4‐KD (4‐KD) cells show higher sensitivity to 5FU than 4‐WT and 4‐Scr. Fig. S8. OVCAR 3 cells show higher sensitivity to cisplatin (CDDP) than OVCAR 4 and Kuramochi. Fig. S9. Kuramochi‐KD (K‐KD) cells show higher sensitivity to cisplatin (CDDP) than K‐WT and K‐Scr. Fig. S10. PTP4A3 silencing does not produce a significant effect in the response of OVCAR 4 cells to cisplatin (CDDP) treatment. Fig. S11. OVCAR 3 cells show higher sensitivity to paclitaxel (PTX) than OVCAR 4 and Kuramochi. Fig. S12. Kuramochi‐KD (K‐KD) cells show higher sensitivity to paclitaxel (PTX) than K‐Scr, however, K‐WT is the most sensitive. Fig. S13. OVCAR 4‐KD (4‐KD) cells show higher sensitivity to paclitaxel (PTX) than 4‐WT and 4‐Scr. Fig. S14. PTP4A3 mRNA expression in OVCAR 4 and Kuramochi cells upon lentiviral‐mediated shRNA knockdown. [file MOL2-19-3427-s001.zip › Figure S5 - revised.tif]

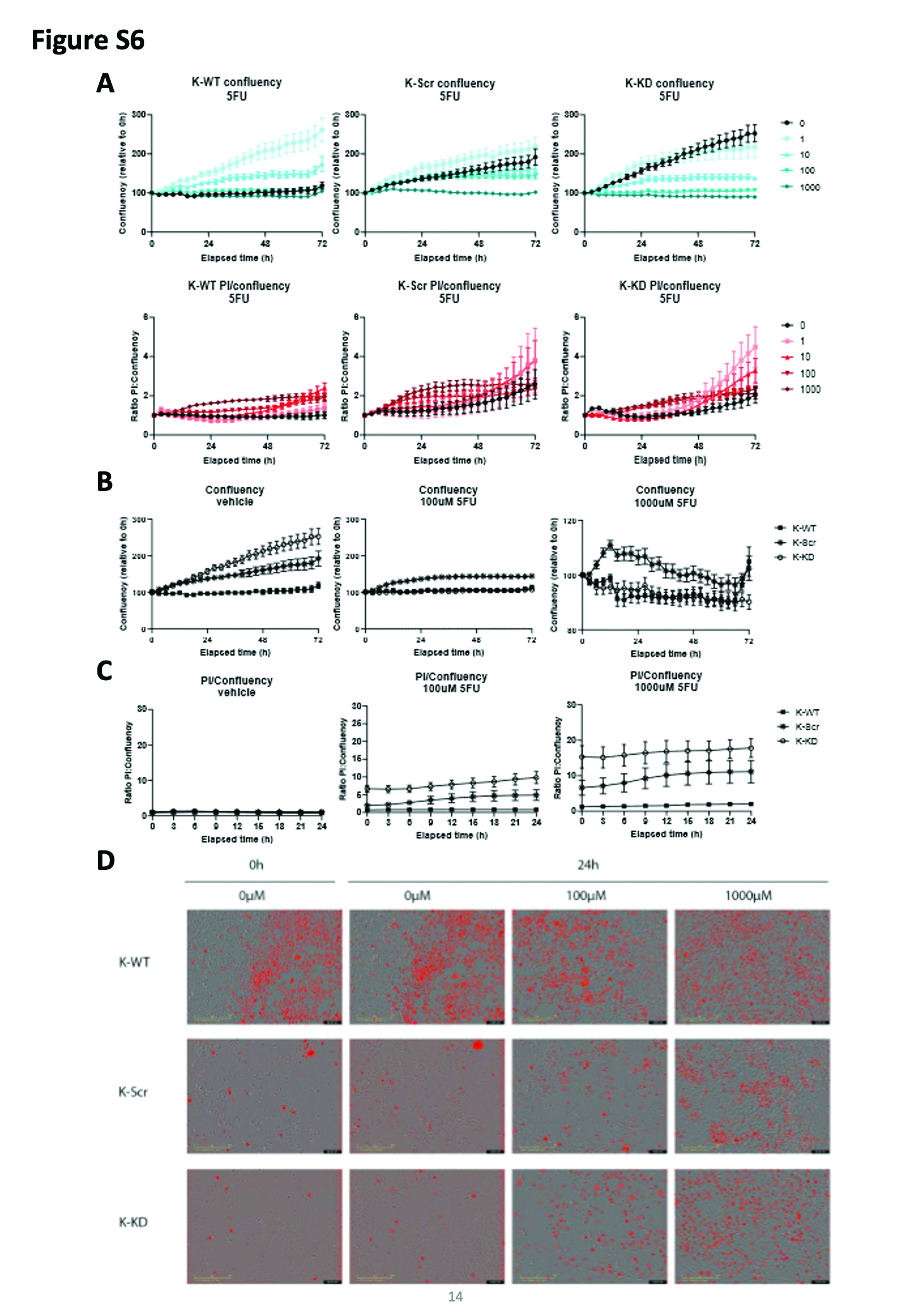

Supplement: Supplementary file 1 — Fig. S1. IC50 curves for JMS‐053, 5FU, CDDP and PTX. Fig. S2. Kuramochi cells show higher sensitivity to the pan‐PTP4A/PRL inhibitor (iPRL) than OVCAR 3 and OVCAR 4 cells. Fig. S3. Kuramochi‐KD (K‐KD) cells show higher sensitivity to PRL inhibitor (iPRL) than K‐Scr cells, however, K‐Scr shows higher resistance than K‐WT cells. Fig. S4. OVCAR 4‐KD (4‐KD) cells show higher sensitivity to the PRL inhibitor (iPRL) than 4‐WT and 4‐Scr cells. Fig. S5. Kuramochi cells show higher sensitivity to 5FU than OVCAR 3 and OVCAR 4. Fig. S6. Kuramochi‐KD (K‐KD) cells show higher sensitivity to 5FU than K‐Scr, however, K‐Scr shows higher resistance than K‐WT. Fig. S7. OVCAR 4‐KD (4‐KD) cells show higher sensitivity to 5FU than 4‐WT and 4‐Scr. Fig. S8. OVCAR 3 cells show higher sensitivity to cisplatin (CDDP) than OVCAR 4 and Kuramochi. Fig. S9. Kuramochi‐KD (K‐KD) cells show higher sensitivity to cisplatin (CDDP) than K‐WT and K‐Scr. Fig. S10. PTP4A3 silencing does not produce a significant effect in the response of OVCAR 4 cells to cisplatin (CDDP) treatment. Fig. S11. OVCAR 3 cells show higher sensitivity to paclitaxel (PTX) than OVCAR 4 and Kuramochi. Fig. S12. Kuramochi‐KD (K‐KD) cells show higher sensitivity to paclitaxel (PTX) than K‐Scr, however, K‐WT is the most sensitive. Fig. S13. OVCAR 4‐KD (4‐KD) cells show higher sensitivity to paclitaxel (PTX) than 4‐WT and 4‐Scr. Fig. S14. PTP4A3 mRNA expression in OVCAR 4 and Kuramochi cells upon lentiviral‐mediated shRNA knockdown. [file MOL2-19-3427-s001.zip › Figure S6.tif]

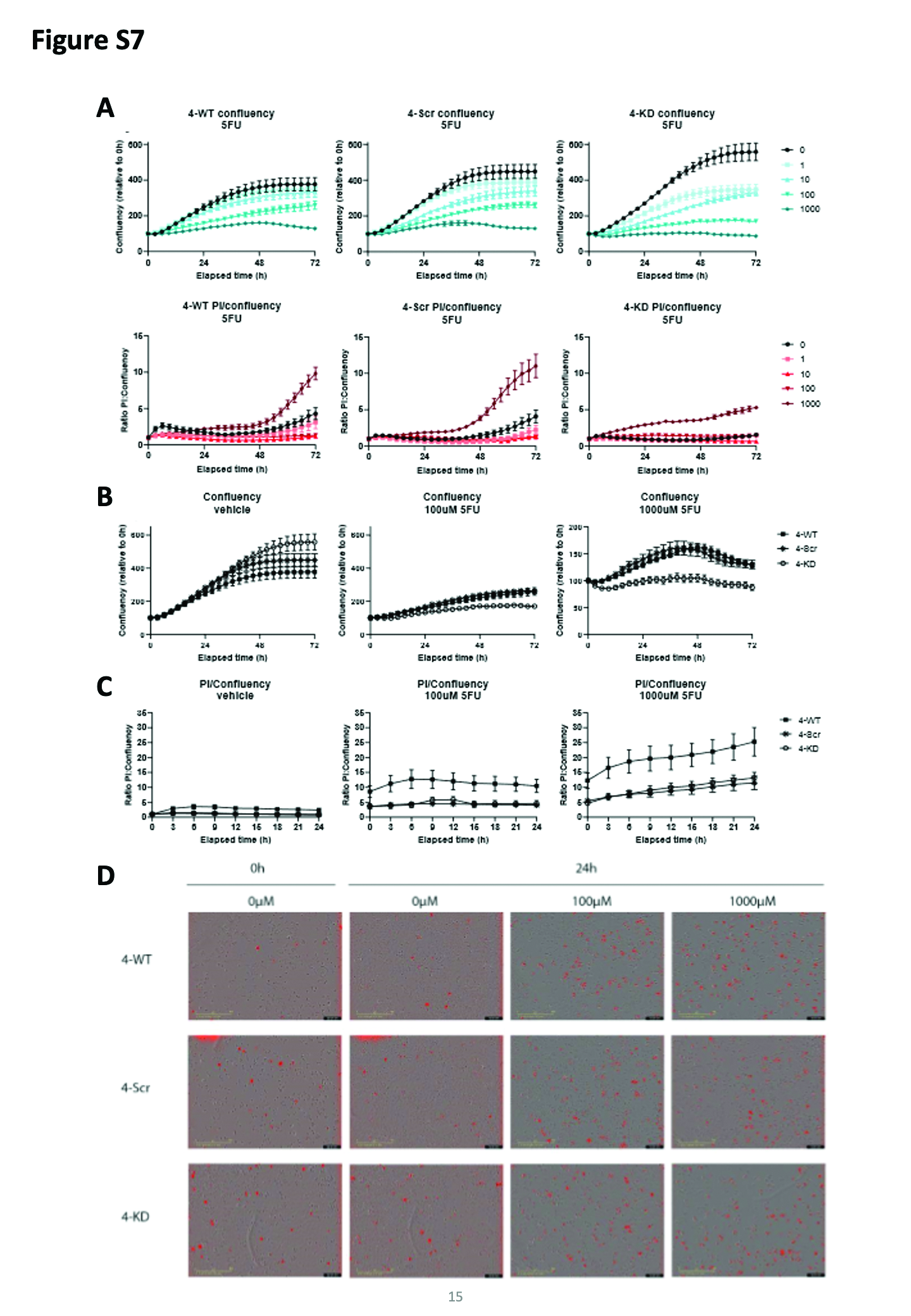

Supplement: Supplementary file 1 — Fig. S1. IC50 curves for JMS‐053, 5FU, CDDP and PTX. Fig. S2. Kuramochi cells show higher sensitivity to the pan‐PTP4A/PRL inhibitor (iPRL) than OVCAR 3 and OVCAR 4 cells. Fig. S3. Kuramochi‐KD (K‐KD) cells show higher sensitivity to PRL inhibitor (iPRL) than K‐Scr cells, however, K‐Scr shows higher resistance than K‐WT cells. Fig. S4. OVCAR 4‐KD (4‐KD) cells show higher sensitivity to the PRL inhibitor (iPRL) than 4‐WT and 4‐Scr cells. Fig. S5. Kuramochi cells show higher sensitivity to 5FU than OVCAR 3 and OVCAR 4. Fig. S6. Kuramochi‐KD (K‐KD) cells show higher sensitivity to 5FU than K‐Scr, however, K‐Scr shows higher resistance than K‐WT. Fig. S7. OVCAR 4‐KD (4‐KD) cells show higher sensitivity to 5FU than 4‐WT and 4‐Scr. Fig. S8. OVCAR 3 cells show higher sensitivity to cisplatin (CDDP) than OVCAR 4 and Kuramochi. Fig. S9. Kuramochi‐KD (K‐KD) cells show higher sensitivity to cisplatin (CDDP) than K‐WT and K‐Scr. Fig. S10. PTP4A3 silencing does not produce a significant effect in the response of OVCAR 4 cells to cisplatin (CDDP) treatment. Fig. S11. OVCAR 3 cells show higher sensitivity to paclitaxel (PTX) than OVCAR 4 and Kuramochi. Fig. S12. Kuramochi‐KD (K‐KD) cells show higher sensitivity to paclitaxel (PTX) than K‐Scr, however, K‐WT is the most sensitive. Fig. S13. OVCAR 4‐KD (4‐KD) cells show higher sensitivity to paclitaxel (PTX) than 4‐WT and 4‐Scr. Fig. S14. PTP4A3 mRNA expression in OVCAR 4 and Kuramochi cells upon lentiviral‐mediated shRNA knockdown. [file MOL2-19-3427-s001.zip › Figure S7.tif]

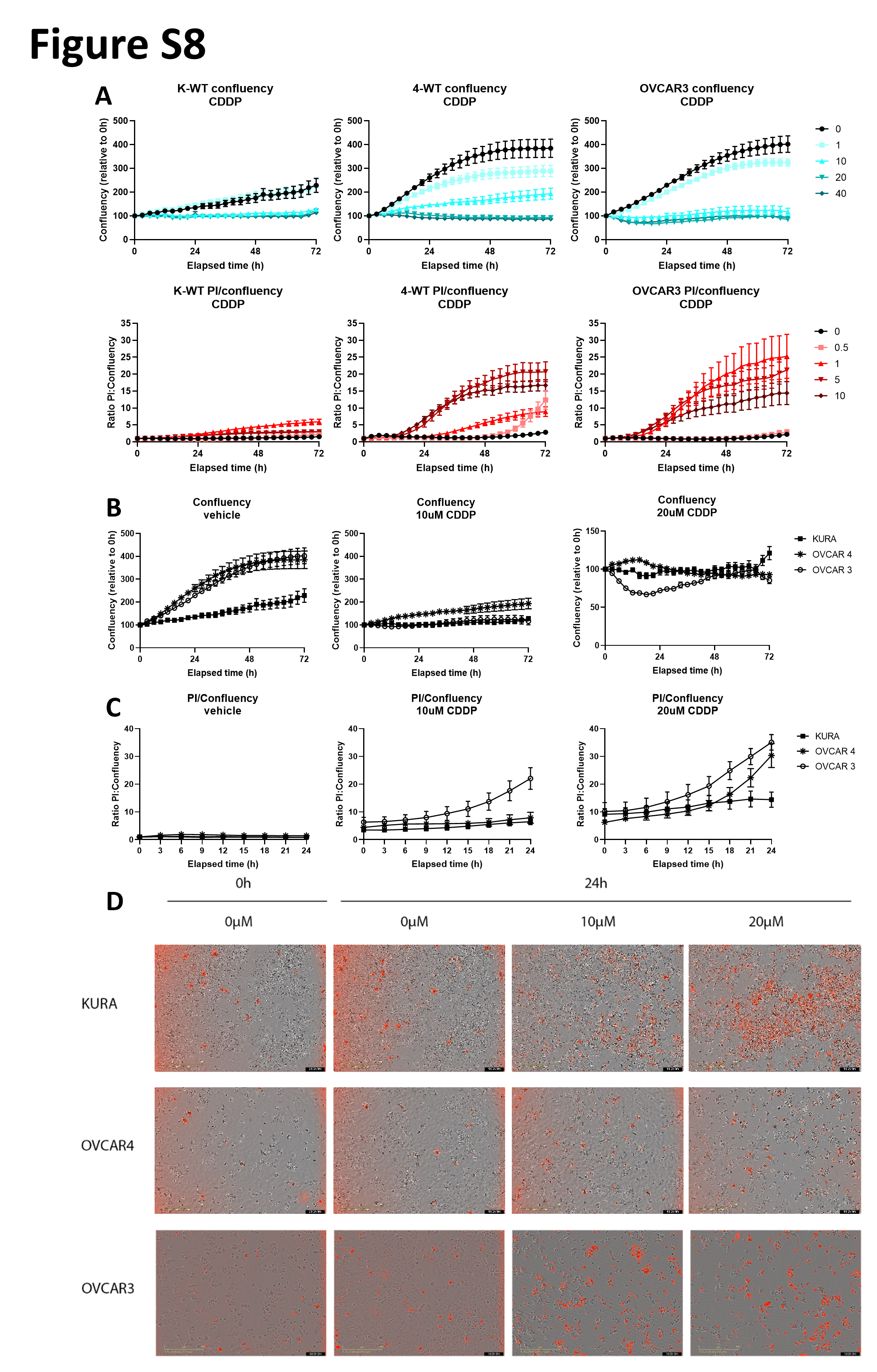

Supplement: Supplementary file 1 — Fig. S1. IC50 curves for JMS‐053, 5FU, CDDP and PTX. Fig. S2. Kuramochi cells show higher sensitivity to the pan‐PTP4A/PRL inhibitor (iPRL) than OVCAR 3 and OVCAR 4 cells. Fig. S3. Kuramochi‐KD (K‐KD) cells show higher sensitivity to PRL inhibitor (iPRL) than K‐Scr cells, however, K‐Scr shows higher resistance than K‐WT cells. Fig. S4. OVCAR 4‐KD (4‐KD) cells show higher sensitivity to the PRL inhibitor (iPRL) than 4‐WT and 4‐Scr cells. Fig. S5. Kuramochi cells show higher sensitivity to 5FU than OVCAR 3 and OVCAR 4. Fig. S6. Kuramochi‐KD (K‐KD) cells show higher sensitivity to 5FU than K‐Scr, however, K‐Scr shows higher resistance than K‐WT. Fig. S7. OVCAR 4‐KD (4‐KD) cells show higher sensitivity to 5FU than 4‐WT and 4‐Scr. Fig. S8. OVCAR 3 cells show higher sensitivity to cisplatin (CDDP) than OVCAR 4 and Kuramochi. Fig. S9. Kuramochi‐KD (K‐KD) cells show higher sensitivity to cisplatin (CDDP) than K‐WT and K‐Scr. Fig. S10. PTP4A3 silencing does not produce a significant effect in the response of OVCAR 4 cells to cisplatin (CDDP) treatment. Fig. S11. OVCAR 3 cells show higher sensitivity to paclitaxel (PTX) than OVCAR 4 and Kuramochi. Fig. S12. Kuramochi‐KD (K‐KD) cells show higher sensitivity to paclitaxel (PTX) than K‐Scr, however, K‐WT is the most sensitive. Fig. S13. OVCAR 4‐KD (4‐KD) cells show higher sensitivity to paclitaxel (PTX) than 4‐WT and 4‐Scr. Fig. S14. PTP4A3 mRNA expression in OVCAR 4 and Kuramochi cells upon lentiviral‐mediated shRNA knockdown. [file MOL2-19-3427-s001.zip › Figure S8 - revised.tif]

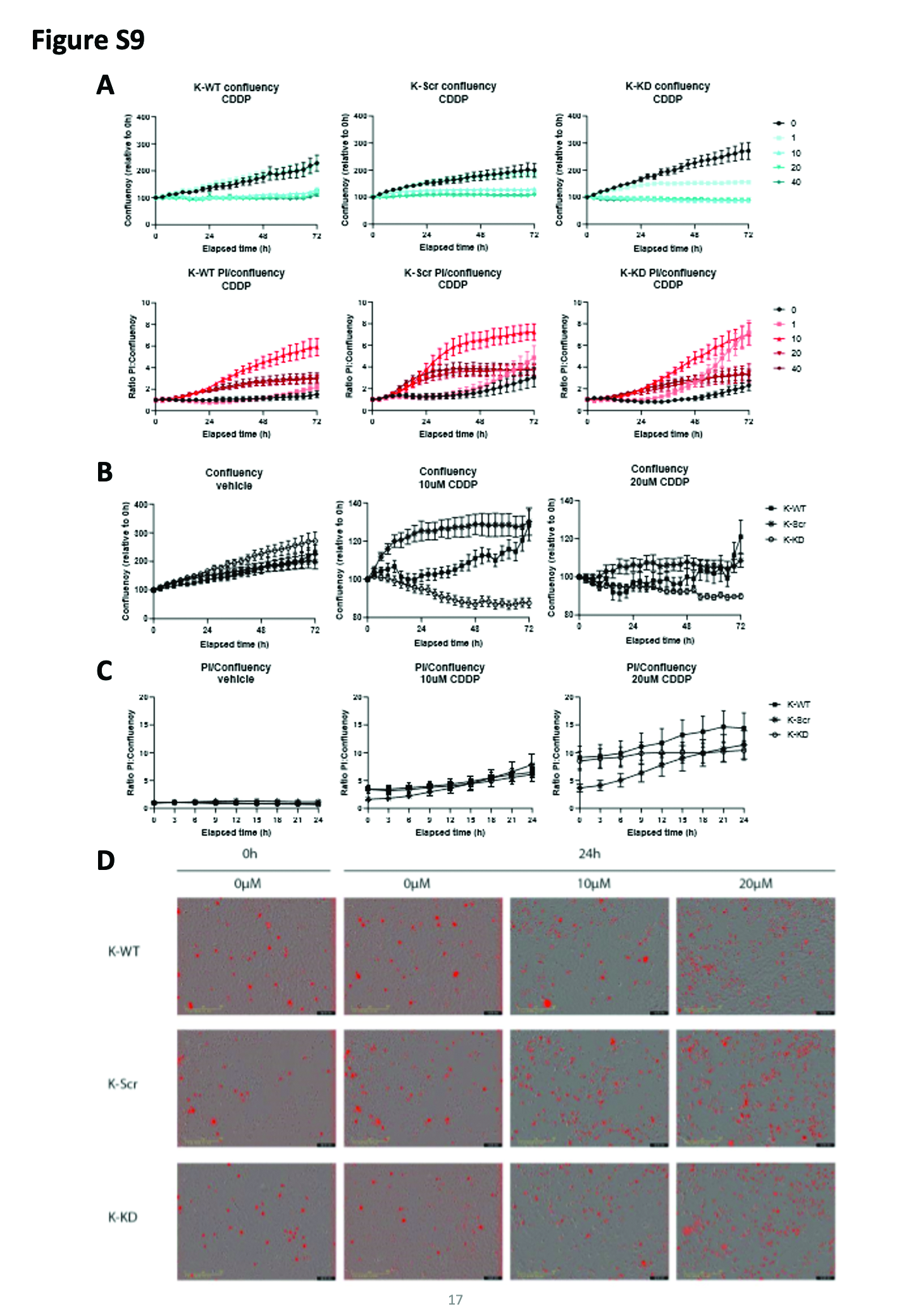

Supplement: Supplementary file 1 — Fig. S1. IC50 curves for JMS‐053, 5FU, CDDP and PTX. Fig. S2. Kuramochi cells show higher sensitivity to the pan‐PTP4A/PRL inhibitor (iPRL) than OVCAR 3 and OVCAR 4 cells. Fig. S3. Kuramochi‐KD (K‐KD) cells show higher sensitivity to PRL inhibitor (iPRL) than K‐Scr cells, however, K‐Scr shows higher resistance than K‐WT cells. Fig. S4. OVCAR 4‐KD (4‐KD) cells show higher sensitivity to the PRL inhibitor (iPRL) than 4‐WT and 4‐Scr cells. Fig. S5. Kuramochi cells show higher sensitivity to 5FU than OVCAR 3 and OVCAR 4. Fig. S6. Kuramochi‐KD (K‐KD) cells show higher sensitivity to 5FU than K‐Scr, however, K‐Scr shows higher resistance than K‐WT. Fig. S7. OVCAR 4‐KD (4‐KD) cells show higher sensitivity to 5FU than 4‐WT and 4‐Scr. Fig. S8. OVCAR 3 cells show higher sensitivity to cisplatin (CDDP) than OVCAR 4 and Kuramochi. Fig. S9. Kuramochi‐KD (K‐KD) cells show higher sensitivity to cisplatin (CDDP) than K‐WT and K‐Scr. Fig. S10. PTP4A3 silencing does not produce a significant effect in the response of OVCAR 4 cells to cisplatin (CDDP) treatment. Fig. S11. OVCAR 3 cells show higher sensitivity to paclitaxel (PTX) than OVCAR 4 and Kuramochi. Fig. S12. Kuramochi‐KD (K‐KD) cells show higher sensitivity to paclitaxel (PTX) than K‐Scr, however, K‐WT is the most sensitive. Fig. S13. OVCAR 4‐KD (4‐KD) cells show higher sensitivity to paclitaxel (PTX) than 4‐WT and 4‐Scr. Fig. S14. PTP4A3 mRNA expression in OVCAR 4 and Kuramochi cells upon lentiviral‐mediated shRNA knockdown. [file MOL2-19-3427-s001.zip › Figure S9.tif]
